# Engineering *Escherichia coli* for high-yielding 2,5-Dimethylpyrazine synthesis from *L*-Threonine by reconstructing metabolic pathways and enhancing cofactors regeneration

**DOI:** 10.1186/s13068-024-02487-4

**Published:** 2024-03-18

**Authors:** Xin-Xin Liu, Yao Wang, Jian-Hui Zhang, Yun-Feng Lu, Zi-Xing Dong, Chao Yue, Xian-Qing Huang, Si-Pu Zhang, Dan-Dan Li, Lun-Guang Yao, Cun-Duo Tang

**Affiliations:** 1 Henan Provincial Engineering Laboratory of Insect Bio-Reactor, Henan International Joint Laboratory of Insect Biology and Henan Key Laboratory of Insect Biology in Funiu Mountain, Nanyang Normal University, 1638 Wolong Road, Nanyang, Henan 473061 People’s Republic of China; 2 Postdoctoral Innovation Practice Base, She Dian Lao Jiu Co. Ltd., 2 Liquor Avenue, Nanyang, Henan 473300 People’s Republic of China; 3 College of Food Science and Technology, Henan Agricultural University, 63 Agricultural Road, Zhengzhou, Henan 450002 People’s Republic of China; 4 Henan Academy of Agricultural Sciences, Zhengzhou, 450002 People’s Republic of China

**Keywords:** 2,5-dimethylpyrazine, Metabolic engineering, Microbial cell factories, Aminoacetone oxidase, Threonine transporter, Whole-cell catalysis

## Abstract

2,5-Dimethylpyrazine (2,5-DMP) is important pharmaceutical raw material and food flavoring agent. Recently, engineering microbes to produce 2,5-DMP has become an attractive alternative to chemical synthesis approach. In this study, metabolic engineering strategies were used to optimize the modified *Escherichia coli* BL21 (DE3) strain for efficient synthesis of 2,5-DMP using *L*-threonine dehydrogenase (*Ec*TDH) from *Escherichia coli* BL21, NADH oxidase (*Eh*NOX) from *Enterococcus hirae*, aminoacetone oxidase (*Sc*AAO) from *Streptococcus cristatus* and *L*-threonine transporter protein (*Ec*SstT) from *Escherichia coli* BL21, respectively. We further optimized the reaction conditions for synthesizing 2,5-DMP. In optimized conditions, the modified strain can convert *L*-threonine to obtain 2,5-DMP with a yield of 2897.30 mg/L. Therefore, the strategies used in this study contribute to the development of high-level cell factories for 2,5-DMP.

**Graphical Abstract:**

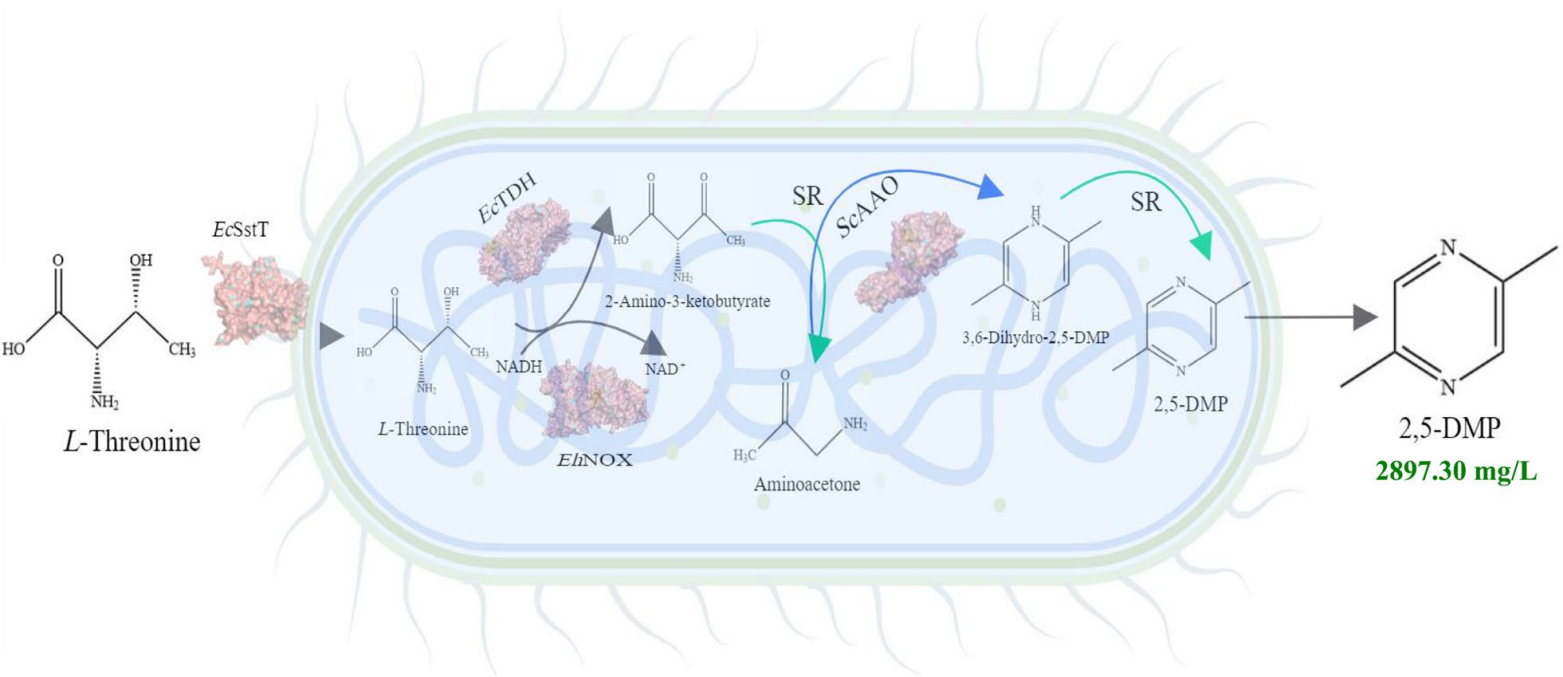

**Supplementary Information:**

The online version contains supplementary material available at 10.1186/s13068-024-02487-4.

## Introduction

2,5-Dimethylpyrazine (2,5-DMP) is a permissible food flavoring agent, which is used in the preparation of flavorings for cocoa [1], coffee beans [2], peanut beans [3, 4], wheat gluten [5], meats [6], nuts [7], potatoes [8], and is also used for fuel [9]. In addition, the 2,5-DMP is used in the pharmaceutical industries, where it is an important raw material for the preparation of 5-methylpyrazine-2-carboxylic acid [10], which is an intermediate in the synthesis of glipizide. This substance is structurally related to the final synthesis of sulfonylurea derivatives and is used in the treatment of type II diabetes. It is also an intermediate in the synthesis of the lipid-lowering drug amoxicillin. *L*-Threonine and *L*-serine had been used to synthesize pyrazines and its derivants [11]. It was found that *L*-serine had been used to synthesize pyrazine, methylpyrazine, 2-ethyl-6methylpyrazine, and 2,6-diethylpyrazine, while *L*-threonine had been used to synthesize 2,5-dimethylpyrazine, 2,6-dimethylpyrazine, trimethylpyrazine, 2-ethyl-3,6-dimethylpyrazine, and 2-ethyl-3,5-dimethylpyrazine [11]. Tomoharu Motoyama et al. demonstrated that 3-ethyl-2,5-dimethylpyrazine can be produced from *L*-threonine by a simple bacterial manipulation system [12]. Ding Y et al. developed an efficient C_3_N-based whole-cell biotransformation system for the synthesis of 2,5-DMP by overexpressing two enzymes TDH and NOX in *Escherichia coli* via the C3N pathway [13]. Yang et al. enhanced enzymatic and non-enzymatic reactions in metabolic pathways by overexpressing TDH and SOAAO genes, and knocked out KBL to block competing branching carbon flow metabolic pathways. Finally, the highest yield transgenic *E. coli* strain reported to date was successfully constructed, and *L*-threonine produced 1682 mg/L 2,5-DMP after 24 h of fermentation reaction [14]. Research has reported that *L*-threonine transporter can promote the transport of *L*-threonine from in vitro to cells [15].

**Scheme 1:**
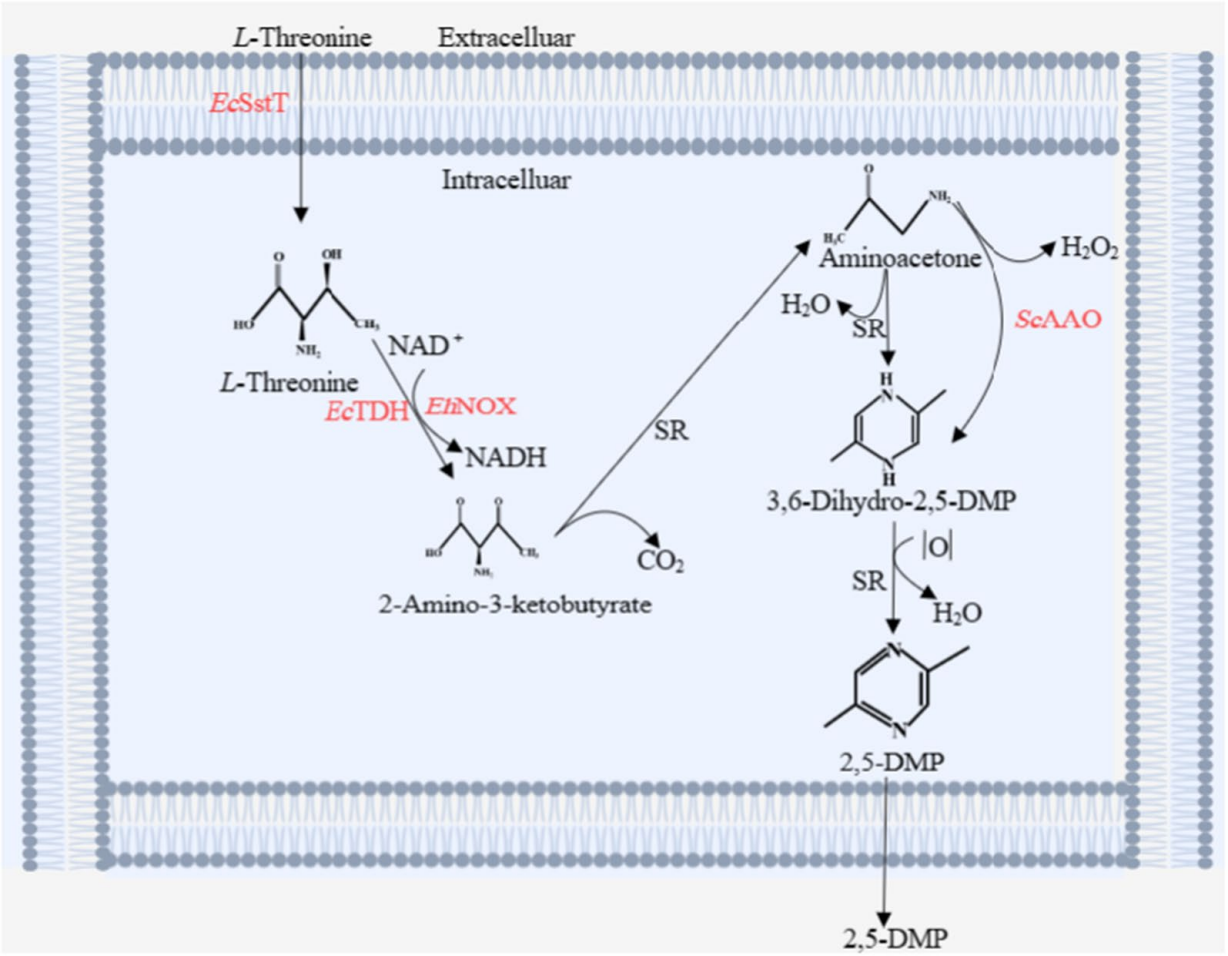
Roadmap for the synthesis of 2,5-DMP using *L*-threonine as a substrate *Ec*SstT–Threonine transporter derived from *Escherichia coli*; *Ec*TDH–*L*-threonine dehydrogenase derived from *Escherichia coli*; *Eh*NOX–NADH oxidase derived from *Enterococcus hirae*; *Sc*AAO–Aminoacetone oxidase derived from *Streptococcus cristatus;* SR-spontaneous reaction

In this work, we attempted to modify the carbon metabolic pathway of *Escherichia coli* based on the theory of metabolic engineering, reduce the formation of by-products, and improve the efficiency of *L*-threonine conversion to 2,5-DMP. Four strategies were employed in this work: (1) choosing the most suitable *L*-threonine dehydrogenase with the highest expression and enzymatic activity, (2) changing the coenzyme cycle and then changing the amount of NAD^+^ and NADH, promote the conversion of *L*-threonine to 2-amino-3-ketobutyrate, (3) enhancing the spontaneous oxidation process of aminoacetone by optimizing the expression of exogenous *Sc*AAO, (4) overexpression of *L*-threonine transporter, so that as much *L*-threonine as possible is transported from outside the cell to inside, thereby promoting the production of 2,5-DMP. The reaction route is shown in Scheme 1. This work may also pave the way for future research investigating natural 2,5-DMP synthesis with high efficiency.

## Material and methods

### Reagents and kits

Restriction enzymes *Xho* I, *Nde* I, *Bam*H I, and *Hind* III were purchased from New England Biolabs, Inc. (NEB). DL 2,000 DNA Marker and PrimeSTAR® HS DNA Polymerase were purchased from TaKaRa (Dalian, China). The NADH and NAD^+^ were purchased from Bontac-bio, Inc. (Shenzhen, China). One Step Cloning Kit was provided by Vazyme (Nanjing, China). Isopropyl β-D-1-thiogalactoside (IPTG) and super-sensitive bacterial preparation kits were purchased from Sangon (Shanghai, China).

### Strains and vectors

The strains and plasmids involved in this study are shown in Additional file 1: Table S1.

### Web server and software

EMBL’s European Bioinformatics Institute (https://www.ebi.ac.uk/) database and Basic Local Alignment Search Tool (https://blast.ncbi.nlm. nih.gov/): protein and gene sequence search; SWISS-MODEL (https://swissmodel.expasy.org/) and RCSB PDB (https://www.rcsb.org/) were selected for multi-template homology modeling of the 3D structures and the results were optimized; AutoDock4.2 was used for molecular docking; PyMOL was employed to observe and analyze the 3D structures and their substrates.

### Instruments and equipment

UNIVERSAL Hood gel imaging system was purchased from Bio-Rad Laboratories, Inc. (Hercules, CA USA) for gel imaging. UV1000 spectrophotometer was purchased from Lairui Scientific Instrument Co, Ltd. (Shanghai, China) for photometric measurements. Protein electrophoresis system PowerPac™/ Mini-PROTEAN® and HC/Mini-PROTEAN® were purchased from Bio-Rad Laboratories, Inc. (Hercules, CA USA) for SDS-PAGE analysis of expression products. A Hypersil C18 column was purchased from Thermo Fisher Scientific Inc. (Waltham, MA USA) for the quantitative analysis of *L*-threonine and 2,5-DMP. Avance III HD 600 MHz and UHPLC-ESI-TOF-MS were purchased from Bruker Corporation (Karlsruhe, Germany) for the qualitative analysis of *L*-threonine and 2,5-DMP.

### Construction of recombinant *E. coli* BL21(DE3)/pACYCDuet-1-*Ec*tdh-*Eh*nox

The database of EMBL (https://www.ebi.ac.uk/) was searched for *L*-threonine dehydrogenase *Ec*TDH (ACI75701.1) derived from *Escherichia coli* and NADH oxidase *Eh*NOX (WP_136382139) derived from *Enterococcus hirae*, respectively, and then specific primers were designed according to the respective gene sequences. Using *E. coli* BL21(DE3) genomic DNA as a template, the gene encoding *L*-threonine dehydrogenase *Ec*TDH was amplified by PCR using *Ec*TDH-F and *Ec*TDH-R primers, and then the target gene and vector pACYCDuet-1 were digested by restriction enzymes *Bam*H I and *Hin*d III, respectively. The products were purified and ligated, and then were transformed into *E. coli* BL21(DE3) receptor cells, the *E. coli* BL21(DE3)/pACYCDuet-1-*Ec*tdh was identified by resistance screening and sequencing. Similarly, an NADH oxidase *Eh*NOX of *Enterococcus hirae* origin was found in EMBL’s European Bioinformatics Institute corresponding genomic sequence number WP_136382139. Upstream and downstream primers for the target gene were designed based on the gene sequence corresponding to *Eh*NOX, and the primer synthesis was performed at Hongxun Biological Technology company (Suzhou, China). The whole genomic DNA of *Enterococcus hirae* was used as the template, and *Eh*NOX-F and *Eh*NOX-R were used as primers for PCR amplification. The PCR product was purified by PCR product purification kit, double digested with restriction enzymes *Nde* I and *Xho* I, ligated to pACYCDuet-1-*Ec*tdh vector which was double digested by the same restriction enzymes, transformed into *E. coli* BL21(DE3) receptor cells, and the recombinant *E. coli* BL21/pACYCDuet-1-*Ec*tdh-*Eh*nox was obtained after resistance screening and double digestion verification.

### Construction of recombinant *E. coli* BL21(DE3)/pETDuet-1-*Sc*aao-*Ec*sstt

The database of EMBL (https://www.ebi.ac.uk/) was searched for the threonine transporter protein *Ec*SstT (CDJ73766.1) from *Escherichia coli* and the aminoacetone oxidase *Sc*AAO (ACA52024.1) from *Streptococcus cristatus*, respectively. Primers were designed by SnapGene and recombinant bacteria were constructed by the one-step cloning method. The *Ec*SstT and *Sc*AAO genes were inserted into the MCS_1_ and MCS_2_ sites of pETDuet-1, respectively. Sequencing and validation were performed on the successfully transformed recombinant bacteria, resulting in the recombinant *E. coli* BL21 (DE3)/pETDuet-1-*Sc*aao-*Ec*sstt.

The pACYCDuet-1-*Ec*tdh-*Eh*nox plasmid and pETDuet-1-*Sc*aao-*Ec*sstt plasmid were simultaneously transformed into *E. coli* BL21(DE3) receptor cells, which were screened for dual resistance to chloramphenicol and ampicillin, and ultimately resulted in the recombinant *E. coli* BL21(DE3)/pACYCDuet-1-*Ec*tdh-*Eh*nox: pETDuet-1-*Sc*aao-*Ec*sstt. The primers designed in this study are shown in Additional file 1: Table S2 and the recombinant *E. coli* cell construction plot is shown in Additional file 1: Fig. 1.

**Fig. 1:**
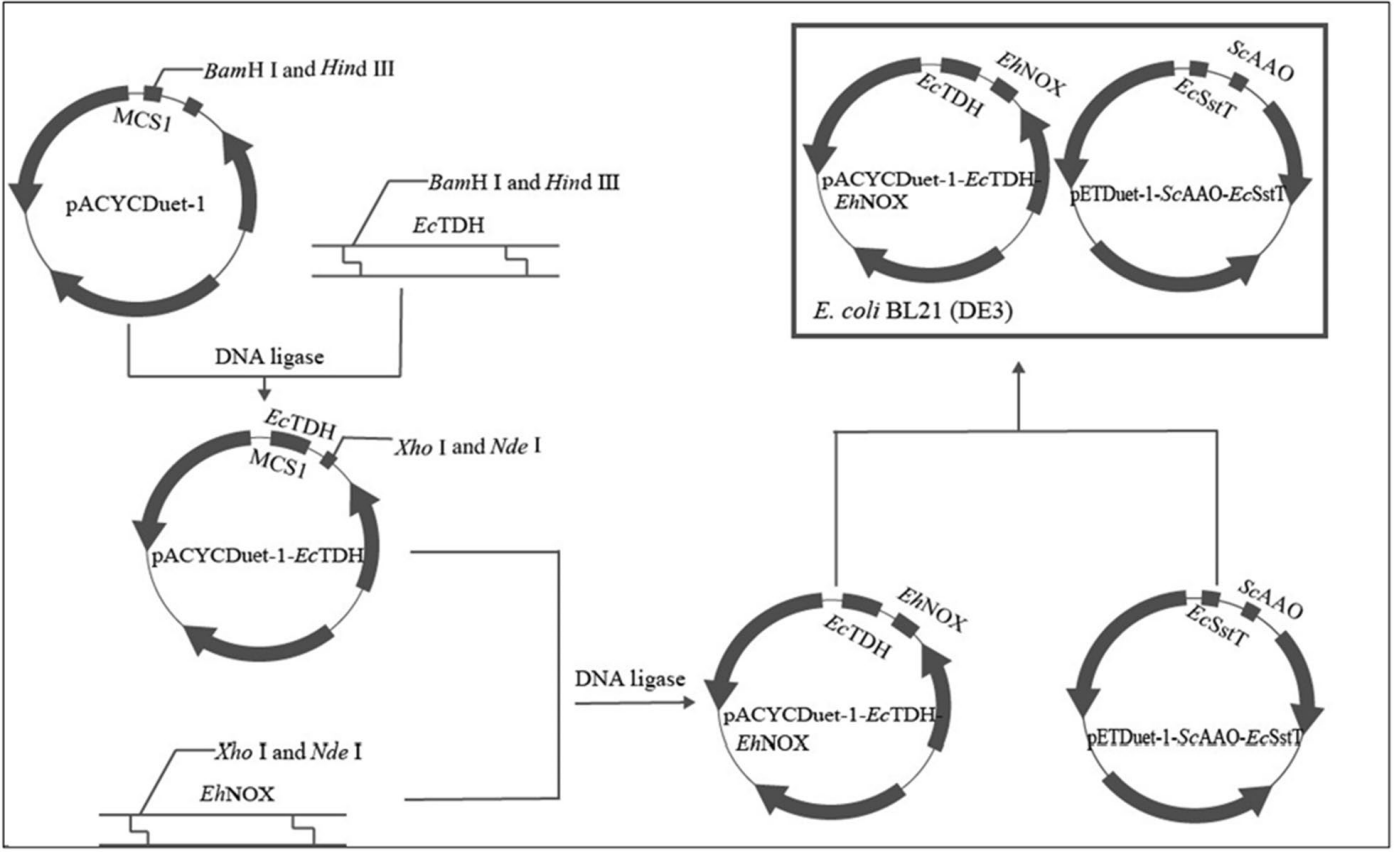
Construction of engineered *E. coli* strain

### Induced expression of recombinant *Escherichia coli*

The induced expression of recombinant *E. coli* was referred to the method of Tang et al. [16, 17]. *E. coli* BL21(DE3)/pACYCDuet-1, *E. coli* BL21(DE3)/pACYCDuet-1-*Ec*tdh, *E. coli* BL21(DE3)/pACYCDuet-1-*Ec*tdh-*Eh*nox, *E. coli* BL21(DE3)/pACYCDuet-1-*Ec*tdh-*Eh*nox: pETDuet-1-*Sc*aao, *E. coli* BL21(DE3)/pACYCDuet-1-*Ec*tdh-*Eh*nox: pETDuet-1-*Sc*aao-*Ec*sstt, *E. coli* BL21(DE3)/pETDuet-1 were inoculated into 5 mL of LB with the corresponding antibiotic resistance. Corresponding antibiotics in LB liquid medium, and finally analyzed by sodium dodecyl sulfate–polyacrylamide gel electrophoresis (SDS-PAGE) as described in Tang et al. [18].

### Enzyme activity analysis

The recombinant *E. coli* cells after induced expression were sonicated and then centrifuged to collect the supernatant to determine the enzyme activity of each recombinant enzyme by the reported methods with slight modifications [14, 19]. The enzyme activity of *Ec*TDH was determined using *L*-threonine as the substrate in a system consisting of 100 mM Tris–HCl, pH 9.0, 100 mM *L*-threonine, and 50 mM NAD^+^. After mixing well and preheating in a metal bath at 30 °C for 5 min, the enzyme solution was added and the change in absorbance at 340 nm was measured within 1 min. The enzyme activity was determined as the amount of enzyme required to generate 1 μmol of NADH in 1 min was 1 IU [20]. The enzyme activity of *Eh*NOX was determined using NADH as substrate in a reaction system containing 10 μM of flavin adenine dinucleotide FAD, 200 μM of NADH, 1 mM of dithiothreitol, 20 mM of citrate-sodium citrate buffer (pH 5.6), and an appropriate amount of enzyme solution [21]. The enzyme activity was defined as the amount of enzyme required to consume 1 μmol NADH in 1 min as 1 IU [22].

### Homology modeling of *Ec*TDH and *Eh*NOX

We used SWISS-MODEL for homology modeling to obtain the precise 3D structures of threonine dehydrogenase and NADH oxidase. The amino acid sequences of two enzymes were used as the query sequence, and the BLAST method was used to search for exogenous crystal structures with high homology (> 60%) with them. These crystal structures are used as templates to model the three-dimensional structures of these two enzymes through multi-template homologous modeling. Next, Validation 3D and PROCHECK were utilized to evaluate the predicted 3D structure.

### Molecular docking

The substrates in molecular docking, as well as the three-dimensional structure of small molecules, were found in PubChem (https://pubchem.ncbi.nlm.nih.gov/). These predicted 3D structures in turn serve as AutoDock 4.2 program docking ligands and receptors. Molecular docking simulations of flexible ligands are performed by genetic algorithms to locate suitable binding sites.

### Biosynthesis of 2,5-DMP from *L*-threonine

A 1 L reaction system containing 100 mM Tris–HCl-NaCl buffer, 60 mmol/L *L*-threonine, 0.75 mmol/L NAD^+^, freeze-dried recombinant *E. coli*, and appropriate amount of glass beads was constructed in a 2 L conical flask. The reaction system was shaken at 37 °C and 220 rpm, and take samples at 24 h. The samples were centrifuged at 12000 rpm for 5 min. The supernatant was taken and filtered with a 0.22 μm filter membrane, and finally the content of 2,5-DMP was detected by HPLC.

### HPLC assay of substrate and products

The 2,5-DMP standard and *L*-threonine were analyzed by HPLC on a Thermo Hypersil C18 column with a detection wavelength of 275 nm and a column temperature of 25℃. The mobile phase A (0.1% formic acid, v/v) and mobile phase B (chromatographic grade methanol) were eluted in a gradient as shown in Additional file 1: Table S3 with a flow rate of 1 mL/min and a running time of 10 min. The 2,5-DMP standard solutions were prepared in ultrapure water at 1.82, 2.27, 3.03, 4.55, and 9.10 mmol/L, filtered through a 0.22 μm microporous filter, then 10 μL was used for HPLC analysis to obtain the peak areas of each concentration of 2,5-DMP, the regression equations and correlation coefficients were obtained by linear fitting. The fermentation broth to be examined was analyzed by HPLC under the same chromatographic conditions, the corresponding sample concentration could be calculated from the regression equation based on the measured peak area.

### ^1^H nuclear magnetic resonance and mass spectrometry analysis

Mass spectrometry conditions are as follows [23]: source: electrospray ion (ESI); source temperature: 120 ℃; mass spectrometry scan mode: positive ion; scan range: 50–1200 m/z; capillary voltage: 2.73–2.75 kV; cone voltage: 40 V; cone gas flow: 50 L h^−1^; desolvation gas flow: 600 L h^−1^; and desolvation temperature: 450 ℃. The ^1^H NMR spectra were recorded on BrukerAvance DPX 250 or BrukerAvance 400 Spec-trometers. Chemical shifts are given in ppm and are referenced to the deuterated solvent signal or to TMS as internal standard and multiplicities are reported as s (singlet), d (doublet), t (triplet), q (quartet) and m (multiplet) [24].

## Results and discussion

### Construction of recombinant *Escherichia coli*

According to the construction method of recombinant *E. coli*, the whole genomic DNA of *E. coli* was used as the template, *Ec*TDH-F and *Ec*TDH-R were used as the primers for PCR amplification, and the amplified products were subjected to agarose gel electrophoresis, as shown in Additional file 1: Figure S1A. The PCR product was purified with the kit, and the purified product and pACYCDuet-1 vector were simultaneously digested by restriction enzymes *Bam*H I and *Hin*d III, and then the digested product was ligated at 16 °C overnight, and the ligation product was transformed into *E. coli* BL21(DE3) receptor cells. After screening for chloramphenicol resistance, the recombinant bacteria were subjected to double enzyme digestion validation. The results were shown in Additional file 1: Figure S1B, the fragment length was about 1100 bp, and the size was consistent with its expected band size. The recombinants verified by PCR and double digestion were sent to Hongxun Biological Technology Company (Suzhou, China) for sequencing and identification, and the gene sequence of *Ec*TDH was obtained, and its amino acid sequence was deduced. The results showed that the sequence of *Ec*TDH and the insertion position on the vector were consistent with the expected results, indicating that the recombinant *E. coli* BL21(DE3)/pACYCDuet-1-*Ec*tdh had been successfully constructed.

Similarly, PCR amplification was carried out using the whole genomic DNA of *Enterococcus hirae* as a template and *Eh*NOX-F and *Eh*NOX-R as primers, and the amplified products were subjected to agarose gel electrophoresis, as shown in Additional file 1: Figure S2A, a clear specific band appeared at about 1400 bp, and the fragment of the PCR product was basically consistent with the expected theoretical length. The PCR product was purified with the kit, and the purified product and pACYCDuet-1-*Ec*tdh vector were simultaneously digested by restriction enzymes *Nde* I and *Xho* I, the ligation product was transformed into *E. coli* BL21(DE3) receptor cells, and then screened for chloramphenicol resistance, and the recombinants were picked up, and then verified by double digestion. The results are shown in Additional file 1: Figure S2B, the fragment length was about 1400 bp, and the size was consistent with its expected band size. The results showed that the sequences of *Ec*TDH and *Eh*NOX and their insertion positions on the vector were consistent with the expected results, indicating that the recombinant bacterium *E. coli* BL21(DE3)/pACYCDuet-1-*Ec*tdh-*Eh*nox had been successfully constructed.

The vector pETDuet-1 was linearized by a one-step cloning method, and the linearized product was subjected to agarose gel electrophoresis, and the results were shown in Additional file 1: Figure S3, which showed a clear specific band around about 5400 bp, and the fragment of the PCR product was basically consistent with the expected theoretical length. Agarose gel electrophoresis of the inserted fragments of the target genes *Ec*SstT and *Sc*AAO were shown in Additional file 1: Figure S4A and S4B, with distinct specific bands appearing around about 1300 bp and 1200 bp, and the sizes were in line with their expected band sizes. The recombinants detected by PCR were validated as shown in Additional file 1: Figure S4C, and their fragment sizes were consistent with the expected results, and were sent to Hongxun Biological Technology Company (Suzhou, China) for sequencing and identification, and the gene sequences of *Ec*SstT and *Sc*AAO were obtained, and their amino acid sequences were deduced. The results showed that the sequences of *Ec*SstT and *Sc*AAO and their insertion positions on the vector were consistent with the expected results, indicating that the recombinant *E. coli* BL21(DE3)/pETDuet-1-*Sc*aao-*Ec*sstt had been successfully constructed.

Finally, the constructed recombinant vectors pACYCDuet-1-*Ec*tdh-*Eh*nox and pETDuet-1-*Sc*aao-*Ec*sstt were transformed into *E. coli* BL21(DE3) receptor cells, which were screened for dual resistance to chloramphenicol and ampicillin, and the recombinants were selected for PCR assay, the results of the assay were shown in Additional file 1: Figure S5. The specific bands of *Ec*TDH, *Eh*NOX, *Ec*SstT, and *Sc*AAO appeared around 1100, 1400, 1300, and 1200 bp, respectively, and the results were as expected, indicating that the recombinant *E. coli* BL21(DE3)/pACYCDuet-1-*Ec*tdh-*Eh*nox: pETDuet-1-*Sc*aao-*Ec*sstt had been successfully constructed.

### Sequence analysis of *Ec*TDH, *Eh*NOX, *Ec*SstT, and *Sc*AAO

The amino acid sequences of the four enzymes postulated above were used to predict the theoretical physicochemical properties by ProtParam (https://web.expasy.org/protparam/) [25]. Their results are shown in Table 1. And predicting the transmembrane structure of the proteins using the TMHMM-2.0 server showed that all the amino acid residues of *Ec*TDH, and *Eh*NOX and *Sc*AAO were extramembrane residues, which indicating that all three proteins were theoretically easy to achieve extracellular secretion expression.

**Table 1:** Sequence analysis of *Ec*TDH, *Eh*NOX, *Ec*SstT, and *Sc*AAO

Enzyme	Theoretical pI	Theoretical molecular weight (Da)	Instability index II	Half-lives in *E. coli* (h)	Half-lives in *yeast* (h)
*Ec*TDH	5.81	37173.94	24.46	> 10	> 20
*Eh*NOX	4.88	49314.96	26.96	> 10	> 20
*Ec*SstT	8.81	43507.72	35.19	> 10	> 20
*Sc*AAO	6.93	42678.90	31.52	> 10	> 20

### Analysis of the catalytic mechanism by molecular docking

**Fig. 2:**
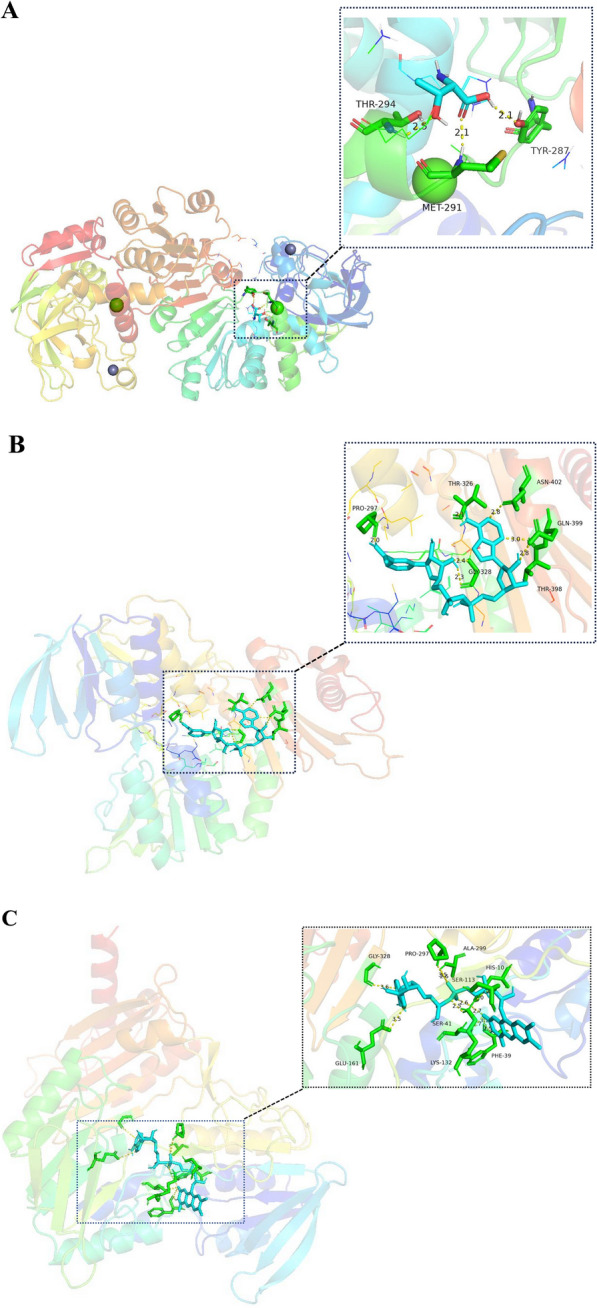
Molecular docking diagram of *Ec*TDH and *Eh*NOX. The molecular docking simulation was performed with flexible ligand by genetic algorithm to locate the suitable binding orientation using AutoDock 4.2 program. These hydrogen bonds are labelled by PyMOL. **A** Docking of the *L*-threonine into the catalytic domain of *Ec*TDH; **B** Docking of the NADH into the catalytic domain of *Eh*NOX; **C** Docking of the FAD into the catalytic domain of *Eh*NOX

To study the catalytic mechanism, AutoDock 4.2 was used to dock the simulated 3D structures of TDH with *L*-Threonine. The docking result was then observed and structurally analyzed using PyMOL [26], as shown in Fig. 2A. The docking results indicated that intermolecular forces were formed between the small molecules and the receptor proteins Tyr287, Met291 and Thr294. The form of this intermolecular force is relatively homogeneous, and all of them are connected by hydrogen bonding [27, 28]. From Fig. 2A, we found that the hydrogen bonds are relatively close to the active site, which is favorable to enhance the binding tightness between the receptor proteins and the small molecules. It has been reported that FAD-dependent NADH oxidase includes two binding domains, a FAD-binding domain and a NAD-binding domain [29, 30]. Based on the above studies, we chose the small molecule NADH and coenzyme FAD to be docked with NOX. From the docking results, the binding energy between small molecule NADH and NOX docking is − 8.2 kcal/mol. Figure 2B shows the results of our docking of small molecule NADH with NOX, from which we can see the formation of hydrogen bonds between small molecule NADH and amino acid residues Pro297, Thr326, Gly328, Thr398, Gln399, Asn402 around the active site. Figure 2C shows the results of molecular docking of the coenzyme FAD with NOX, and the binding energy of the docking between the two is − 10.5 kcal/mol. From the docking results, the binding energy of NOX docked to FAD was lower than that of NOX docked to NADH, suggesting that the FAD-dependent NADH oxidase originating from *Enterococcus hirae* binds more tightly to FAD. As can be seen in Fig. 2C, hydrogen bonds are formed between small molecules of NADH and amino acid residues His10, Phe39, Ser41, Ser113, Lys132, Glu161, Gly328, Pro297, and Ala299, resulting in a more stable structure.

### Induced expression and characterization of engineered *E. coli* strains

**Fig. 3:**
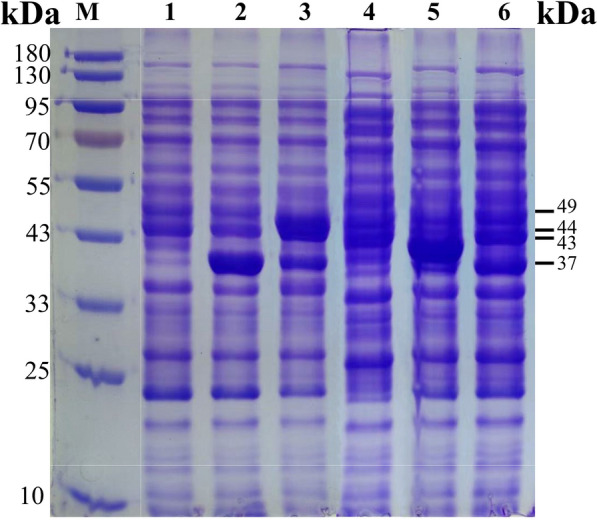
SDS-PAGE analysis of recombinant *Escherichia coli* expression products. M, PageRuler Prestained Protein Ladder; 1, expression products of *E. coli* BL21(DE3)/pACYCDuet-1; 2, crude expression product of *E. coli* BL21(DE3)/pACYCDuet-1-*Ectdh*; 3, crude expression product of *E. coli* BL21(DE3)/pACYCDuet-1-*Ectdh*-*Ehnox*; 4, crude expression product of *E. coli* BL21(DE3)/pETDuet-1; 5, crude expression product of *E. coli* BL21(DE3)/pACYCDuet-1-*Ectdh*-*Ehnox*: pETDuet-1-*Sc*aao; 6, crude expression product of *E. coli* BL21(DE3)/pACYCDuet-1-*Ectdh*-*Ehnox*: pETDuet-1-*Sc*aao-*Ec*sstt

The engineered *E. coli* strains were subjected to induced expression according to the low temperature and low concentration inducer method, and the lysate supernatant was analyzed by SDS-PAGE. The results of SDS-PAGE analysis are shown in Fig. 3. The lysate supernatant of *E. coli* BL21(DE3)/pACYCDuet-1-*Ec*tdh-*Eh*nox: pETDuet-1-*Sc*aao-*Ec*sstt showed obvious specific bands at about 37, 43, 44 and 49 kDa, which were consistent with the theoretical molecular masses of *Ec*TDH, *Sc*AAO, *Ec*SstT and *Eh*NOX, respectively, indicating that soluble expression of all four target enzymes had been successfully achieved.

### Enzyme activity analysis of *Ec*TDH and *Eh*NOX

The enzyme activity analysis of *Ec*TDH and *Eh*NOX of each recombinant are shown in Table 2. The results showed that *Ec*TDH and *Eh*NOX could be expressed simultaneously using dual plasmid functional expression system of pACYCDuet-1 and pETDuet-1, and a microbial cellular factory for multi-stage enzyme reaction was successfully constructed with the ability to catalyze the synthesis of *L*-threonine to 2,5-DMP.

**Table 2:** Enzyme activity of TDH and NOX from different engineered *E. coli*

Strains	Enzyme activity(IU/mL)	
	TDH	NOX
*E. coli* BL21/pACYCDuet-1	0.31	0.26
*E. coli* BL21/pACYCDuet-1-*Ectdh*	25.32	0.32
*E. coli* BL21/pACYCDuet-1-*E*c*tdh-Ehnox*	24.82	2.83
*E. coli* BL21/pACYCDuet-1-*E*c*tdh-Ehnox*: pETDuet-1-*Sc*aao	23.20	2.49
*E. coli* BL21/pACYCDuet-1-*E*c*tdh-Ehnox*: pETDuet-1-*Sc*aao-*Ec*sstt	18.55	2.38
*E. coli* BL21/pETDuet-1	0.27	0.35

### HPLC analysis of 2,5-DMP and *L*-Thr standards

The standard 2,5-DMP was analyzed by HPLC on a Thermo Hypersil C18 column. Under this analytical condition, the retention time of 2,5-DMP was about 6.62 min, and the different concentration values of 2,5-DMP were linearly fitted to the corresponding peak areas, the standard curve of 2,5-DMP obtained is shown in Additional file 1: Figure S6, which yielded that the concentration of 2,5-DMP. The corresponding regression equation was *y* = 2062.4*x* + 354.29, and the correlation coefficient was 0.9978. The results showed that the concentration of 2,5-DMP showed a good linear correlation with the peak area under this HPLC analytical condition and concentration range. Therefore, 2,5-DMP can be analyzed accurately and quantitatively using this method. The HPLC analysis of the substrate *L*-threonine under the same conditions showed that the retention time of the substrate was about 2.69 min. Under the same analytical conditions, the difference between the retention time of *L*-threonine and the retention time of 2,5-DMP was about 3.5 min, and the results are shown in Additional file 1: Figure S7A and S7B, respectively, which indicated that a good separation between *L*-threonine and 2,5-DMP could be achieved under the chromatographic conditions, and this chromatographic method can be used for the analysis and identification of fermentation broth.

### Engineered *E. coli* whole-cell-catalyzed synthesis of 2,5-DMP from *L*-threonine

**Fig. 4:**
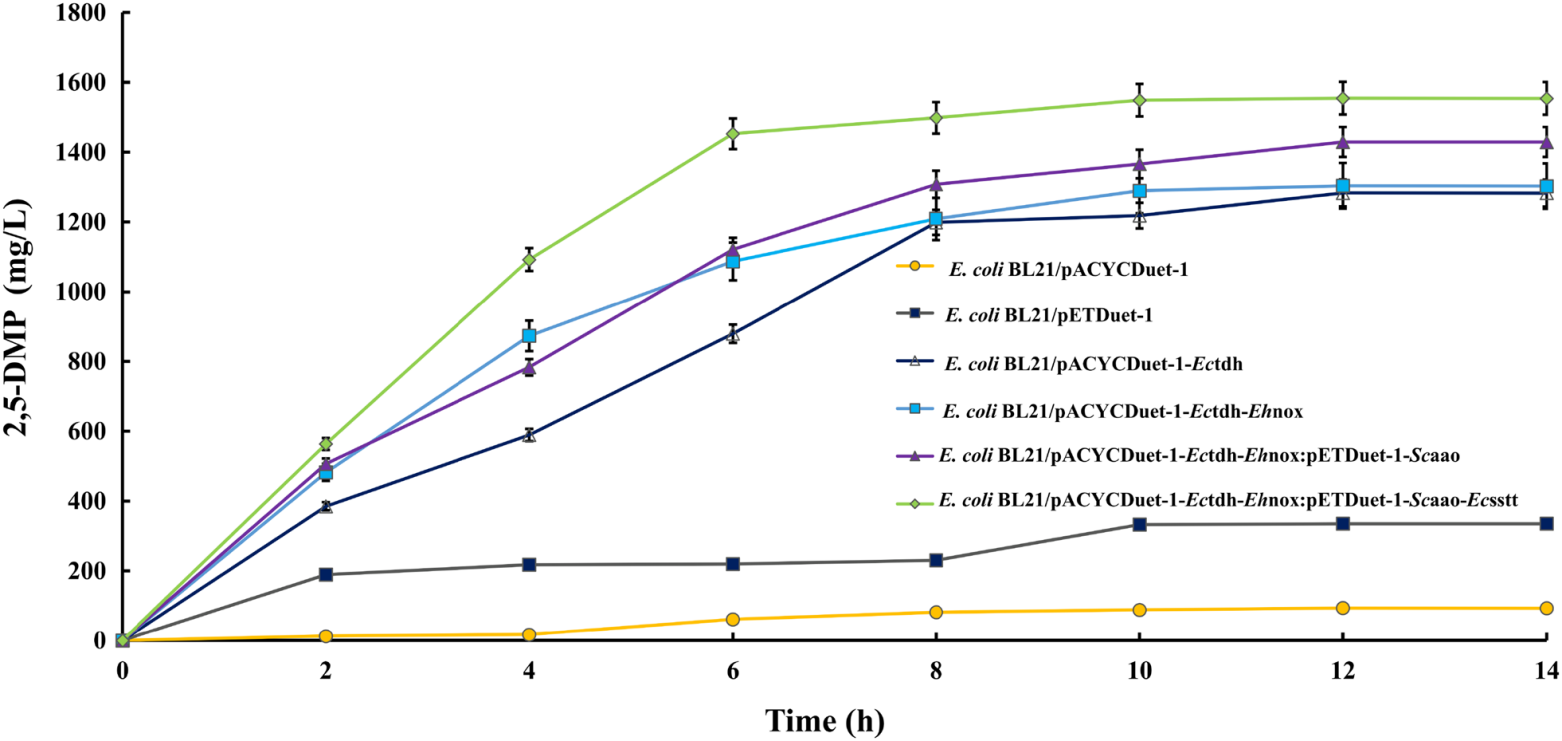
The substrate *L*-threonine to product of 2,5-DMP reaction process diagram. The product was sampled at reaction time 2, 4, 6, 8,10,12 and 14 h to detect by HPLC. The reaction time in horizontal coordinate and the production of 2,5-DMP in vertical coordinate were, respectively

The whole-cell catalytic biotransformation of *L*-threonine was carried out according to the whole-cell catalytic method. The fermentation broth was centrifuged and analyzed by HPLC according to the above method, and the retention times were about 6.62 and 2.69 min (Additional file 1: Figure S8), which were in agreement with the retention times of the 2,5-DMP and *L*-threonine under the same analytical conditions, indicating that some of *L*-threonine had been converted to 2,5-DMP under the catalytic conditions, and their catalytic reaction progress curves were shown in Fig. 4**.** The reaction basically reached equilibrium at 12 h, and the yield of 2,5-DMP basically no longer varied with time. This result indicated that the empty vector carried by *Escherichia coli* has background expression, but the yield was extremely low. When *L*-threonine dehydrogenase was introduced, the production of 2,5-DMP synthesized by *L*-threonine significantly increased. On the basis of overexpressing *L*-threonine dehydrogenase, NADH oxidase was introduced, which continuously converted NADH to NAD^+^, promoted the conversion of *L*-threonine to 2-amino-3-ketobutyrate, and then promoted the generation of 2,5-DMP, resulting in a 1.6% increase in yield. Further overexpression of aminoacetone oxidase on the previous basis resulted in the conversion of aminoacetone to 3,6-dihydro-2,5-dimethylpyrazine, which resulted in a 9.6% yield increasement compared to the former. Considering that *L*-threonine has to enter from the outside to the inside of the cell, and it has been shown that the introduction of the threonine transporter protein can facilitate the transport of *L*-threonine from extracellular to intracellular pathways, the present study introduced the threonine transporter protein on the basis of the above [31], to obtain higher yield, and when the threonine transporter protein was introduced, the yield was increased by 8.8% compared with the former. The above results suggest that overexpression of *L*-threonine transporter protein can indeed enhance the yield of 2,5-DMP by allowing extracellular *L*-threonine to be transported intracellularly [32]. The results of the significance of difference analysis are shown in Additional file 1: Figure S11. The results showed that our constructed recombinant bacteria had an extremely significant difference in the ability to produce 2,5-DMP compared with the empty vector. We conducted a significant difference analysis between the final constructed recombinant bacteria and two blank groups, and the results were both *p* ≤ 0.001, indicating extremely significant differences. This result indicates that the recombinant bacteria we ultimately constructed play an extremely important role in the synthesis of 2,5-dimethylpyrazine.

### Qualitative analysis of fermentation broth components

The results of NMR and MS analysis of the substance in the retention time of 6.62 min are shown in Additional file 1: Figure S9A and S9B, respectively. The mass charge ratio of the substance is 109.0760. According to the mass-to-charge ratio, the molecular formula of the substance may be C_6_H_9_N_2_^+^ (M + H)^+^, indicating that the substance may be C_6_H_8_N_2_ (2,5-DMP) with one H^+^ removed. The results of NMR and MS analysis of the substance in the retention time of 2.69 min are shown are shown in Additional file 1: Figure S10A and S10B, respectively. The mass charge ratio of the product is 120.0661. According to the mass-to-charge ratio, the molecular formula of the product may be C_4_H_10_O_3_ (M + H)^+^, indicating that the product may be C_4_H_9_O_3_ (*L*-Thr) with one H^+^ removed.

### Effect of pH on the whole-cell catalyzed synthesis of 2,5-DMP from *L*-threonine

The sensitivity of enzymes to acids and bases is one of the biological characteristics of enzymes, which is manifested in the fact that the activity and stability of enzymes are very easily affected by the environmental pH. And the environmental pH has a more significant effect on enzyme activity, and the reason why pH affects enzyme activity is most likely due to the fact that it alters the dissociation state of the relevant groups in the active site of enzymes. The dissociation state of the active group on the enzyme molecule is most suitable for the binding of the enzyme to the substrate at the optimal pH value. The ionic nature and ionic strength of the buffer system may also have an effect on the enzyme reaction. In addition, the pH value has a great influence on the enzyme activity, but also on the stability of the enzyme, too high or too low pH will change the conformation of the enzyme active center, or even change the structure of the whole enzyme molecule, leading to enzyme inactivation.

**Fig. 5:**
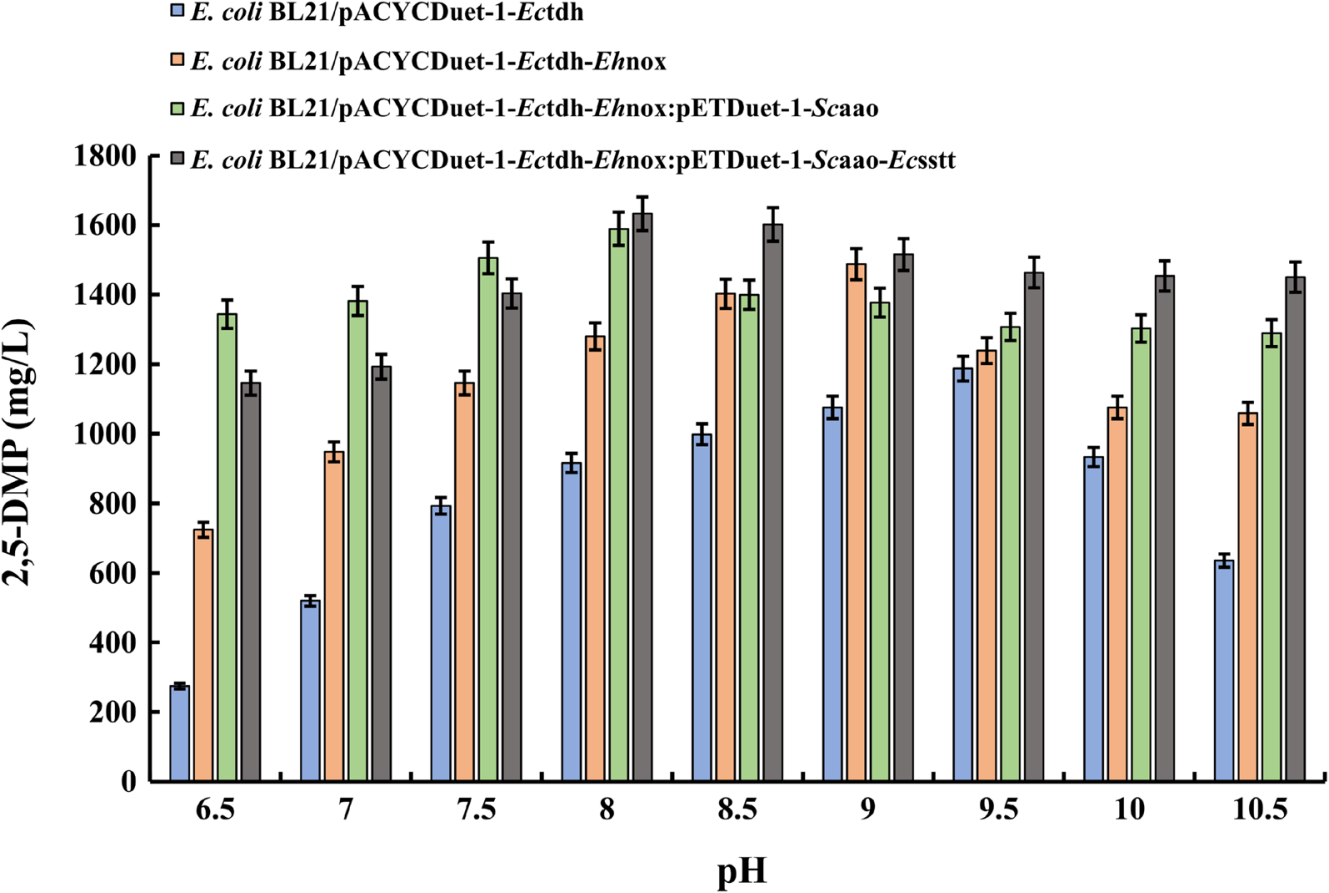
Effect of buffers at different pH on different engineered *E. coli* whole-cell-catalyzed *L*-threonine synthesis of 2,5-DMP. The initial pH of the reaction was set in the range of 6.5–10.5 to explore the influence of different pH values on the amount of products generated

To investigate the effect of the reaction environment pH on the whole-cell synthesis of 2,5-DMP, we controlled the reaction temperature at 35 °C, the reaction time at 12 h, and set the buffer pH to the range of 6.5–10.5, respectively, to investigate the changes in the yield of the product 2,5-DMP in this pH range. The results, as shown in Fig. 5, showed that during the whole-cell-catalyzed reaction, since the optimal pH of various enzymes was not the same, they would interact with each other when two proteins or even three or four proteins were co-expressed, which resulted in different yields of 2,5-DMP produced by each engineered *E. coli* strain under different pH conditions. When threonine dehydrogenase was expressed alone, its optimal pH was 9.5, and its 2,5-DMP production could reach 1187.13 mg/L, while the four enzymes were expressed simultaneously, the optimal pH for fermentation reaction was 8.0, and 2,5-DMP production could reach 1632.69 mg/L, which was a 37.5% yield increasement compared with that of single gene expression. These results indicated that the pH variations in the reaction system of the enzyme affected the stability and the activity of the enzyme. When two enzymes or even multiple enzymes act together, due to the different optimal reaction pH of each enzyme, when reacting in different pH environments, the secondary bonds between each other will interact with each other, such as hydrogen bonding, and ionic, and covalent and other interactions [33, 34]. The results of the significance of difference analysis are shown in Additional file 1: Figure S12. The results showed significant differences among the groups, indicating that the ability of different recombinant bacteria to produce 2,5-DMP under different pH conditions varied greatly, and further indicating that the ability of different recombinant bacteria to produce 2,5-DMP was greatly affected by pH. After further introducing NADH oxidase, we conducted a significant difference analysis with recombinant bacteria overexpressing threonine dehydrogenase alone, and the result was *p* ≤ 0.05, indicating significant differences between the groups. After further introduction of aminoacetone oxidase and the combination of aminoacetone oxidase and threonine transporter protein, significant differences were analyzed in the synthesis of 2,5-dimethylpyrazine with strains overexpressing threonine dehydrogenase alone. The result was *p* ≤ 0.001, indicating extremely significant differences. The above results further indicate that recombinant bacteria overexpressing NADH oxidase, aminoacetone oxidase, and threonine transporter proteins are greatly affected by pH during the synthesis of 2,5-dimethylpyrazine.

### Effect of substrate addition on the whole-cell catalyzed synthesis of 2,5-DMP from *L*-threonine

In the whole-cell catalyzed synthesis of 2,5-DMP from *L*-threonine, there are several branches to synthesize different products during the reaction with *L*-threonine as the substrate, *L*-threonine generates α-ketobutyrate under the action of threonine deaminase and glycine under the action of threonine aldolase. In the process of generating 2,5-DMP through multistage enzymatic reaction under the action of threonine dehydrogenase, there also exist intermediate products for branching reaction such as 2-amino-3-ketobutyrate to generate glycine, amino acetone to generate aminopropan-2-ol, and so on. The above indicated that the synthesis of 2,5-DMP from *L*-threonine is an incomplete reaction process, therefore the consumption of substrate threonine varies.

**Fig. 6:**
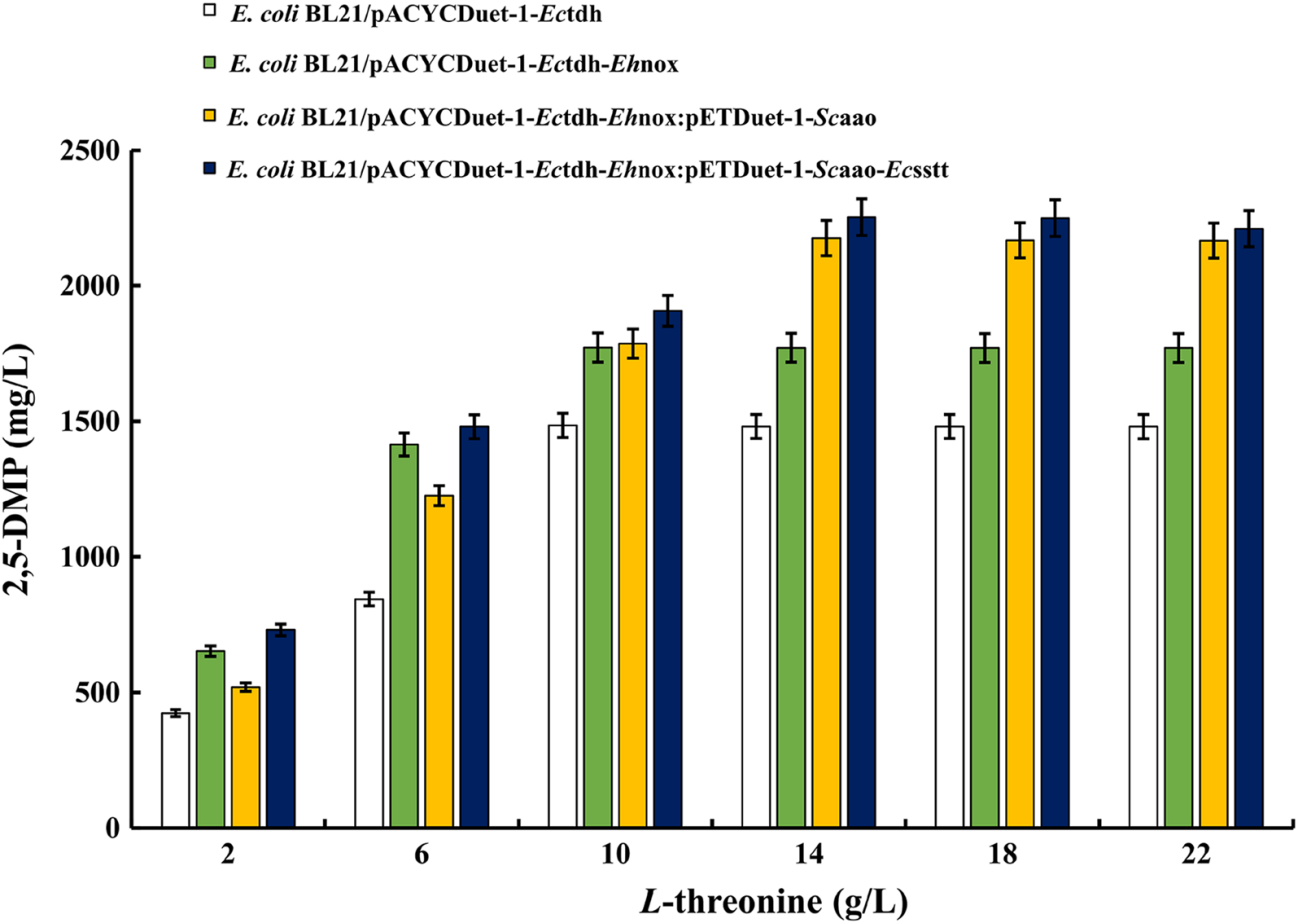
Effects of different substrate addition levels on *L*-threonine synthesis of 2,5-DMP catalyzed by different engineered *E. coli* whole cells. Under the optimal reaction pH condition, the effects of different substrate addition amounts on the product yield were determined, and the substrate addition amounts were 2, 6, 10, 14, 18, 22 g/L, respectively

To investigate the effect of the addition of the substrate *L*-threonine on the synthesis of 2,5-DMP, the reaction temperature was controlled at 35 °C, the reaction time was controlled at 12 h under different optimal pH conditions set for each engineered *E. coli* strain, and the whole-cell catalytic reaction was carried out under the conditions of substrate *L*-threonine addition of 2, 6, 10, 14, 18 and 22 g/L, respectively, and the samples were detected by HPLC assay. And the amount of production was calculated, and the results are shown in Fig. 6. When TDH and TDH- NOX were co-expressed, the optimal substrate addition was 10 g/L, when three and four proteins were co-expressed, the optimal substrate addition could be up to 14 g/L, and its 2,5-DMP production was as high as 2252.05 mg/L. The above results indicated that the efficiency of *L*-threonine transport from extracellular to intracellular was improved when the threonine transporter protein was introduced, which in turn led to a higher yield of 2,5-DMP. The above results verified that threonine transporter proteins can facilitate *L*-threonine transport from extracellular to intracellular, which is consistent with the results of Wang et al. [35]. We analyzed the results of Fig. 6 for significance of differences, as shown in Additional file 1: Figure S13, which showed that there were no significant differences between the groups. This result suggests that the ability to generate 2,5-DMP among the recombinant bacteria is less affected by the amount of substrate added. After overexpressing NADH oxidase, aminoacetone oxidase, and threonine transporter protein, a significant difference analysis was conducted compared to overexpressing threonine dehydrogenase alone, with a result of *p* ≥ 0.05. This further indicates that the effect of substrate addition on the synthesis of 2,5-dimethylpyrazine by each recombinant bacterium is relatively small.

### Effect of temperature on the whole-cell catalyzed synthesis of 2,5-DMP from *L*-threonine

In the process of whole-cell catalysis, the temperature will affect the reaction rate of the enzyme and the physiological state of the bacterium, which in turn will affect the synthesis of the products. In addition, the stability of the enzyme also affects the optimal temperature of whole-cell catalysis, and the higher the temperature, the lower the stability and the easier the enzyme is inactivated. Therefore, choosing the appropriate temperature for whole-cell catalysis is crucial for the synthesis of 2,5-DMP.

**Fig. 7:**
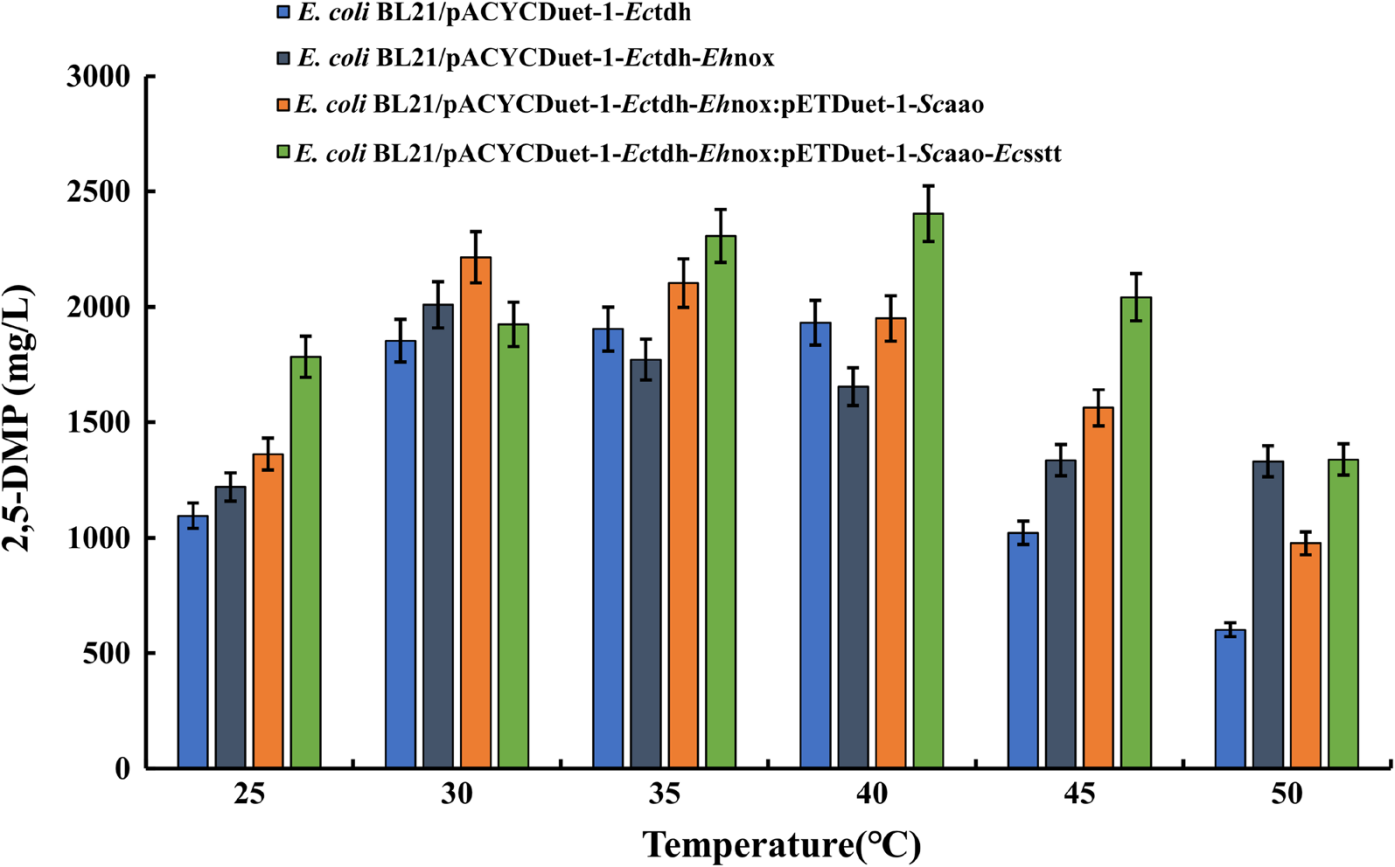
Effects of different temperatures on whole cell-catalyzed *L*-threonine synthesis of 2,5-DMP by different engineered *E. coli*. Under the conditions of the optimal amount of substrate added, and the influence of temperature on the yield of 2,5-DMP was explored under the optimum pH condition. The temperature was set within 25–50 ℃

In this study, the effect of different reaction temperatures on the whole-cell-catalyzed synthesis of 2,5-DMP from *L*-threonine was investigated based on the optimal pH and optimal substrate addition of different engineered *E. coli* strain, with a controlled reaction time of 12 h, and at reaction temperatures of 25, 30, 35, 40, 45, and 50 °C, respectively. The samples were analyzed according to the HPLC assay for detecting 2,5-DMP and the amount of 2,5-DMP produced was calculated. The results are shown in Fig. 7, which indicates that the optimal reaction temperature was 40 °C when TDH was expressed alone and when TDH, NOX, *Sc*AAO, and SstT were co-expressed, whereas the optimal reaction temperature was 30 °C when TDH and NOX were co-expressed and when TDH, NOX, and *Sc*AAO were co-expressed. In optimized conditions, the engineered *E. coli* strain can convert *L*-threonine to obtain 2,5-DMP with a yield of 2472.52 mg/L and a carbon atom yield of 41.3%. This result indicates that different enzymes have different reaction temperatures, and when multiple enzymes undergo co-expression, they affect each other, making the optimal reaction temperatures of different organisms different. The temperature of the external environment affects the changes in enzyme activity, which in turn affects the yield of whole-cell catalyzed reactions. This is attributed to the fact that in this case, the action of disulfide bonds can have some effect on the enzyme structure and can cause changes in the structure of the enzyme active site from an optimal to a non-optimal configuration for substrate binding. The results suggest that the active site of the enzyme determines the effect of temperature on enzyme activity and that the evolution of the active site may be limited by temperature dependence [36, 37]. We analyzed the results of Fig. 7 for significance of differences, and the results as shown in Additional file 1: Figure S14, indicated that there was no significant difference between the groups. We conducted a significant difference analysis between recombinant bacteria overexpressing NADH oxidase, aminoacetone oxidase, and threonine transporter protein, as well as recombinant bacteria overexpressing threonine dehydrogenase alone. The result was *p* ≥ 0.05, indicating that there was no significant difference between them. This result suggests that the ability of recombinant bacteria to generate 2,5-DMP is less affected by temperature. Under these optimal fermentation conditions, the initial pH of the reaction was 8.0, the amount of substrate *L*-threonine added was 14 g/L, and the temperature was 40 ℃. We expanded the reaction in a 1 L reaction system. After 24 h of fermentation, the yield of 2,5-DMP reached 2897.30 mg/L. We will construct the recombinant bacterium *E coli* BL21(DE3)/pACYCDuet-1-*Ec*tdh-*Eh*nox: pETDuet-1-*Sc*aao-*Ec*sstt was used to construct a 50 mL reaction system in a 250 mL flask. The system was incubated at a pH of 8.0, a substrate *L*-threonine addition of 14 g/L, and a temperature of 40 °C. After 12 h of fermentation, the production titer was 2472.52 mg/L. Under the same conditions, we constructed a 1 L reaction system in a 2 L flask, and after 24 h of reaction, the production titer of 2,5-dimethylpyrazine was 2897.30 mg/L. So far, the highest reported titer of 2,5-dimethylpyrazine was 1682 mg/L when the recombinant bacteria constructed by Yang et al. [14]. Reacted for 24 h with an *L*-threonine addition of 9.21 g/L. The above results showed that our constructed recombinant bacterium elevated the titer of 2,5-dimethylpyrazine by 1215.30 mg/L compared with the highest 2,5-dimethylpyrazine that has been reported. This result further suggests that the introduction of NADH oxidase by means of genetic engineering can promote the cycling of coenzymes, change the value of NADH/NAD^+^, and further promote the conversion of *L*-threonine to *L*-2-aminolevulinic acid. When we further exogenously express the threonine transporter protein can make *L*-threonine transported from extracellular to intracellular as much as possible, more *L*-threonine can be consumed to synthesize 2,5-dimethylpyrazine during the whole reaction process, which in turn can make the titer of 2,5-dimethylpyrazine of the whole reaction increase, and also provide some theoretical basis for the large-scale production of 2,5-dimethylpyrazine in the industry.

## Conclusions

2,5-DMP is a metabolite of the threonine metabolic pathway and is widely used in the food and pharmaceutical industries. However, 2,5-DMP is currently synthesized mainly by chemical methods, which is often accompanied by a number of problems. Therefore, most food and pharmaceutical companies have turned to the biosynthesis of 2,5-DMP. However, the production of 2,5-DMP by biosynthesis is insufficient for industrial applications due to its extremely low efficiency. In this study, we obtained the highest synthetic yield of 2,5-DMP reported to date. Under the optimal fermentation reaction conditions, the initial pH of the reaction was 8.0, the amount of substrate *L*-threonine added was 14 g/L, and the temperature was 40 ℃, the constructed 1 L fermentation reaction could yield up to 2897.30 mg/L of 2,5-DMP after 24 h. The constructed microbial cell engineering has the potential to produce higher yields of 2,5-DMP and apply it to industrial production, laying the foundation for future research on natural 2,5-DMP production.

### Supplementary Information

**Additional file 1: Figure S1.** The agarose gel electrophoresis analysis for PCR products of *Ec*TDH and double enzyme digestion verification of recombinant *E. coli* BL21(DE3)/pACYCDuet-1-*Ec*tdh. **Figure S2.** The agarose gel electrophoresis analysis for PCR products of *Eh*NOX and double enzyme digestion verification of recombinant *E. coli *BL21(DE3)/pACYCDuet-1-*Ec*tdh-*Eh*nox. **Figure S3.** Analysis of PCR products of vector pETDuet-1 by agarose gel electrophoresis. **Figure S4.** The agarose gel electrophoresis analysis for PCR products of *Ec*SstT and *Sc*AAO and universal primer validation of recombinant *E. coli* BL21(DE3)/pETDuet-1-*Sc*aao-*Ec*sstt. **Figure S5.** The universal primer validation of recombinant *E. coli* BL21(DE3)/pACYCDuet-1-*Ec*tdh-*Eh*nox: pETDuet-1-*Sc*aao-*Ec*sstt. **Figure S6.** Correlation analysis of 2,5-DMP with peak area. **Figure S7A.** HPLC analysis of substrate *L*- threonine standard. **B** HPLC analysis of 2,5-DMP. **Figure S8.** HPLC analysis catalytic products. **Figure S9A.** NMR analysis of products in fermentation broths. **B** MS analysis of products in fermentation broths. **Figure S10A.** NMR analysis of substrates in fermentation broths. **B** MS analysis of substrates in fermentation broths. **Figure S11.** The results in Figure 4 were analyzed for significance of differences. **Figure S12.** The results in Figure 5 were analyzed for significance of differences. **Figure S13.** The results in Figure 6 were analyzed for significance of differences. **Figure S14.** The results in Figure 7 were analyzed for significance of differences. **Table S1.** Strains and plasmids used in this study. **Table S2.** Primers used in this study. **Table S3.** HPLC gradient elution of the product 2,5-DMP.

## Data Availability

The data sets used during the current study are available from the corresponding author on reasonable request.
