# Supplementary material for: Engineering Escherichia coli for high-yielding 2,5-Dimethylpyrazine synthesis from L-Threonine by reconstructing metabolic pathways and enhancing cofactors regeneration
Source: Biotechnol Biofuels Bioprod. 2024 Mar 18;17:44. doi: 10.1186/s13068-024-02487-4 (PMC10949639; doi:10.1186/s13068-024-02487-4)
Supplement: Supplementary file 1 — Additional file 1: Figure S1. The agarose gel electrophoresis analysis for PCR products of EcTDH and double enzyme digestion verification of recombinant E. coli BL21(DE3)/pACYCDuet-1-Ectdh. Figure S2. The agarose gel electrophoresis analysis for PCR products of EhNOX and double enzyme digestion verification of recombinant E. coli BL21(DE3)/pACYCDuet-1-Ectdh-Ehnox. Figure S3. Analysis of PCR products of vector pETDuet-1 by agarose gel electrophoresis. Figure S4. The agarose gel electrophoresis analysis for PCR products of EcSstT and ScAAO and universal primer validation of recombinant E. coli BL21(DE3)/pETDuet-1-Scaao-Ecsstt. Figure S5. The universal primer validation of recombinant E. coli BL21(DE3)/pACYCDuet-1-Ectdh-Ehnox: pETDuet-1-Scaao-Ecsstt. Figure S6. Correlation analysis of 2,5-DMP with peak area. Figure S7A. HPLC analysis of substrate L- threonine standard. B HPLC analysis of 2,5-DMP. Figure S8. HPLC analysis catalytic products. Figure S9A. NMR analysis of products in fermentation broths. B MS analysis of products in fermentation broths. Figure S10A. NMR analysis of substrates in fermentation broths. B MS analysis of substrates in fermentation broths. Figure S11. The results in Figure 4 were analyzed for significance of differences. Figure S12. The results in Figure 5 were analyzed for significance of differences. Figure S13. The results in Figure 6 were analyzed for significance of differences. Figure S14. The results in Figure 7 were analyzed for significance of differences. Table S1. Strains and plasmids used in this study. Table S2. Primers used in this study. Table S3. HPLC gradient elution of the product 2,5-DMP. [file 13068_2024_2487_MOESM1_ESM.docx]

**Engineering *Escherichia coli* for High-Yielding 2,5-Dimethylpyrazine Synthesis from *L*-Threonine by Reconstructing Metabolic Pathways and Enhancing Cofactors Regeneration**

**Figure S1. The agarose gel electrophoresis analysis for PCR products of *Ec*TDH and double enzyme digestion verification of recombinant *E. coli* BL21(DE3)/pACYCDuet-1-*Ectdh*.**


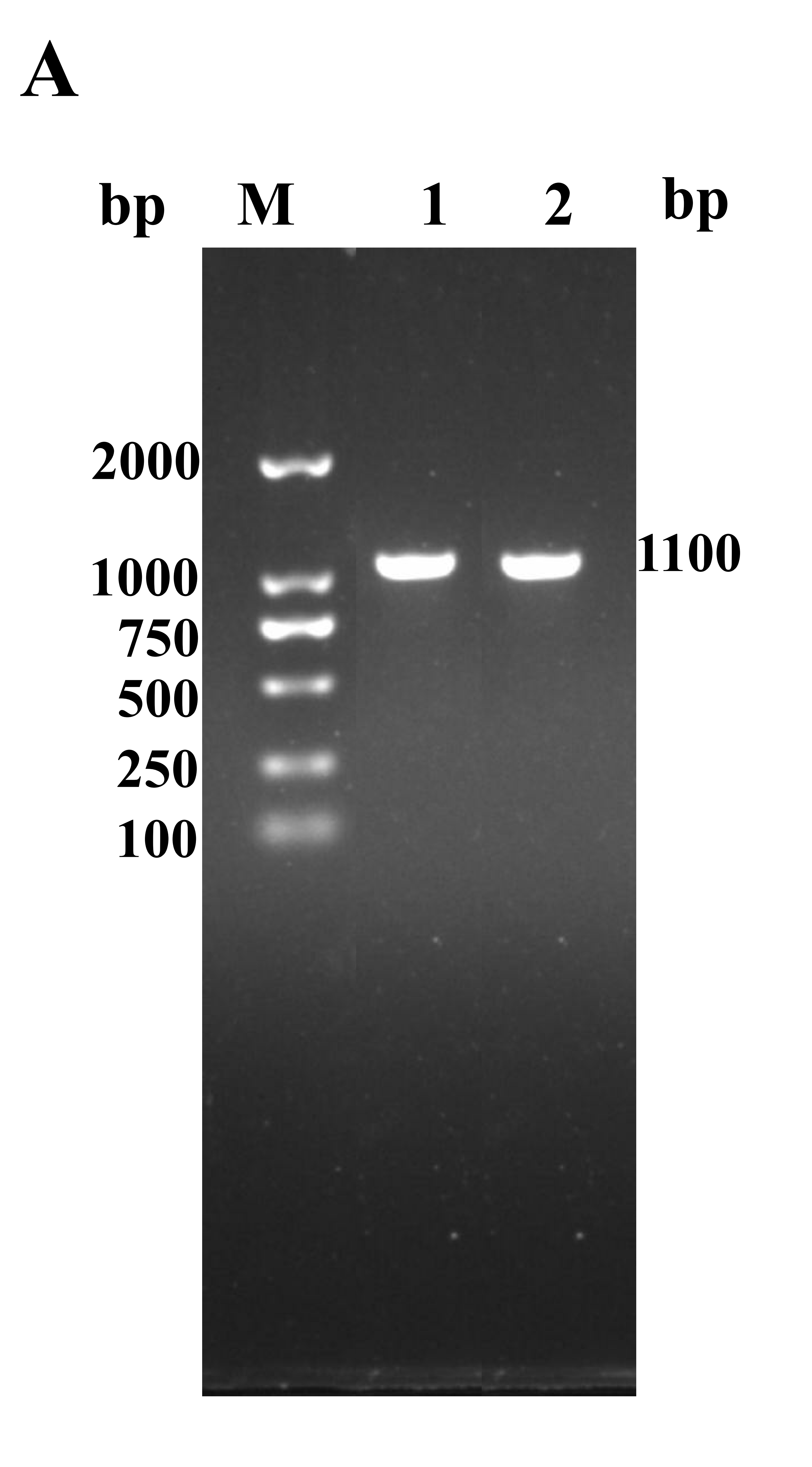

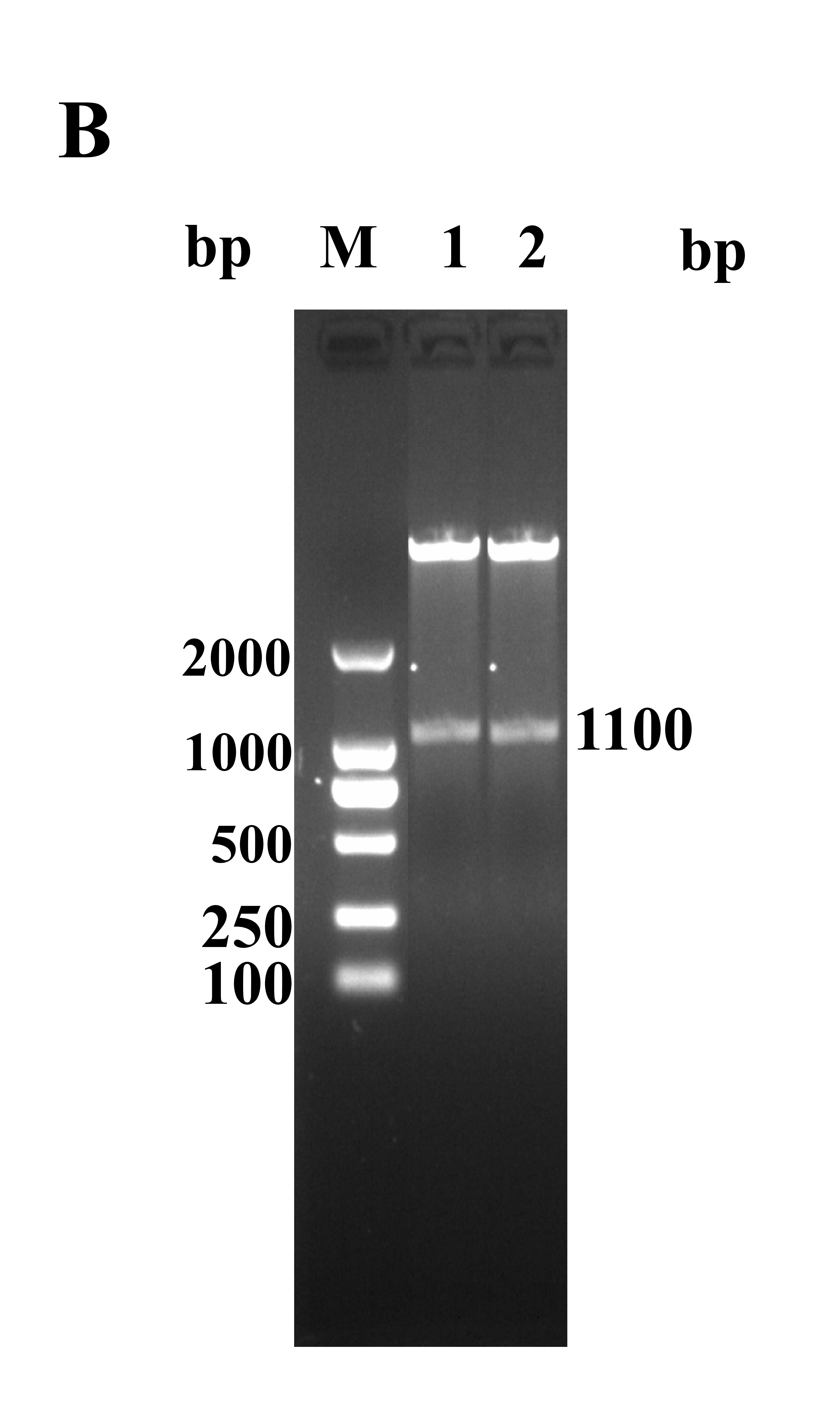


(A) PCR detection. Lane M, DNA marker; lane 1-2, PCR products from different strains of *E. coli* BL21(DE3)/pACYCDuet-1-*Ectdh*; (B) Double digestion detection. Lane M, DNA marker; lane 1-2, Double digestion products of *E. coli* BL21（DE3）/pACYCDuet-1-*Ectdh* by *Bam*H Ⅰ and *Hin*d Ⅲ.

**Figure S2. The agarose gel electrophoresis analysis for PCR products of *Eh*NOX and double enzyme digestion verification of recombinant *E. coli* BL21(DE3)/pACYCDuet-1-*Ectdh*-*Ehnox***


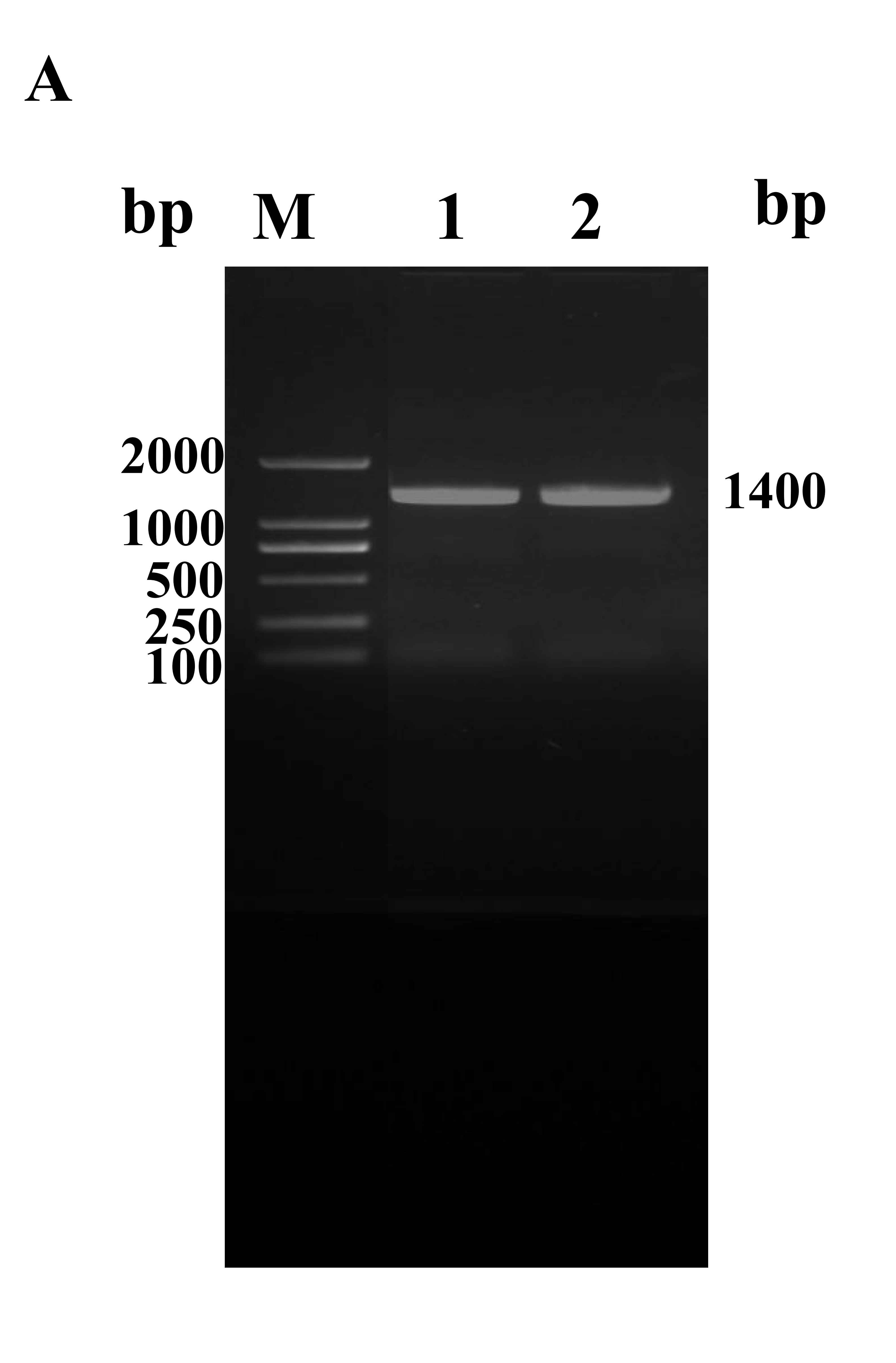

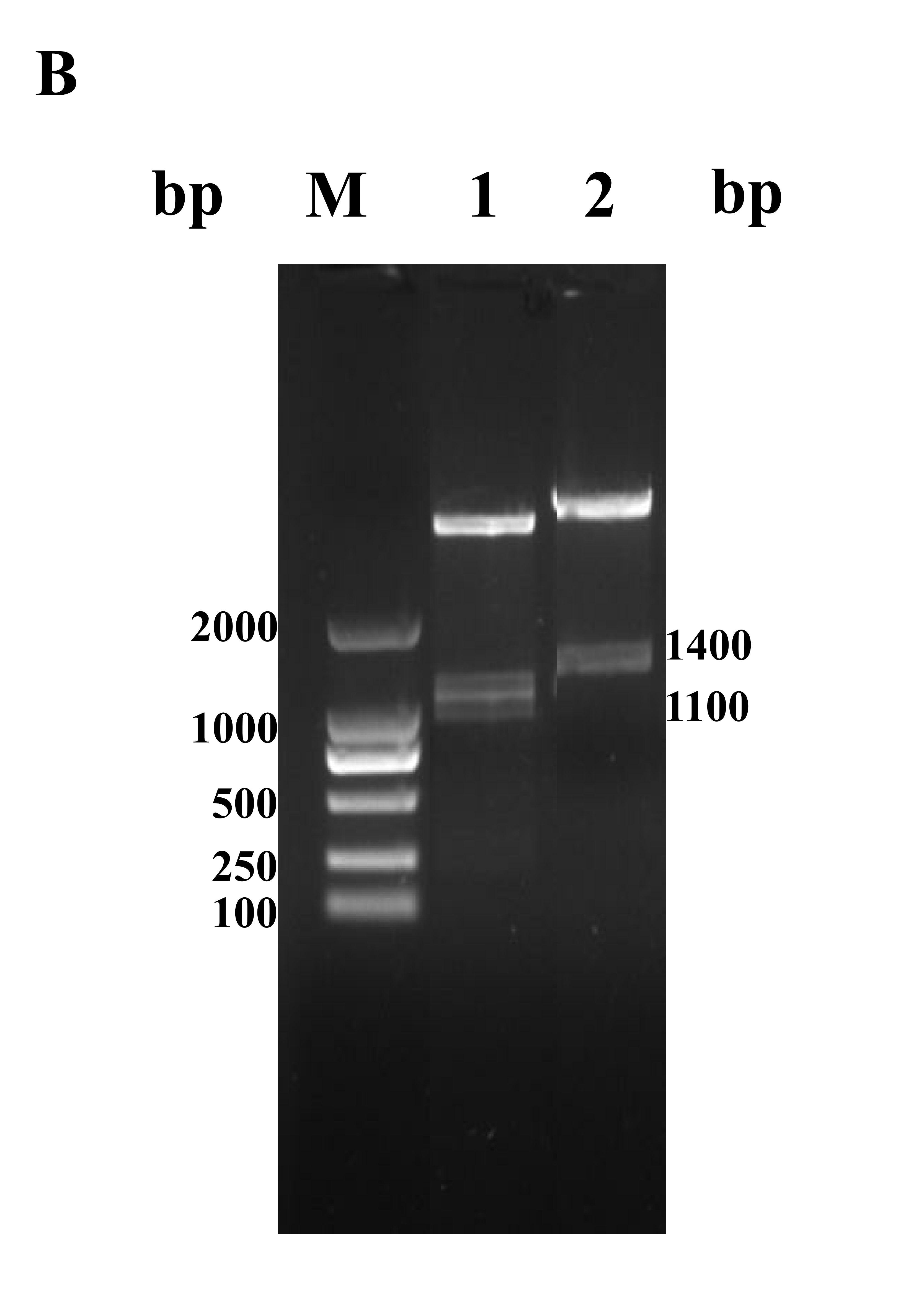


(A) PCR detection. Lane M, DNA marker; lane 1-2, PCR products from different strains of *E. coli* BL21(DE3)/pACYCDuet-1-*Efnox*; (B) Double digestion detection. Lane M, DNA marker; lane 1, Double digestion products of *E. coli* BL21（DE3）/pACYCDuet-1-*Ectdh*-*Efnox* by *Bam*H Ⅰ and *Hin*d Ⅲ; lane 2, Double digestion products of *E. coli* BL21（DE3）/pACYCDuet-1-*Ectdh*-*Efnox* by *Nde* Ⅰ and *Xho* Ⅰ

**Figure S3. Analysis of PCR products of vector pETDuet-1 by agarose gel electrophoresis.**


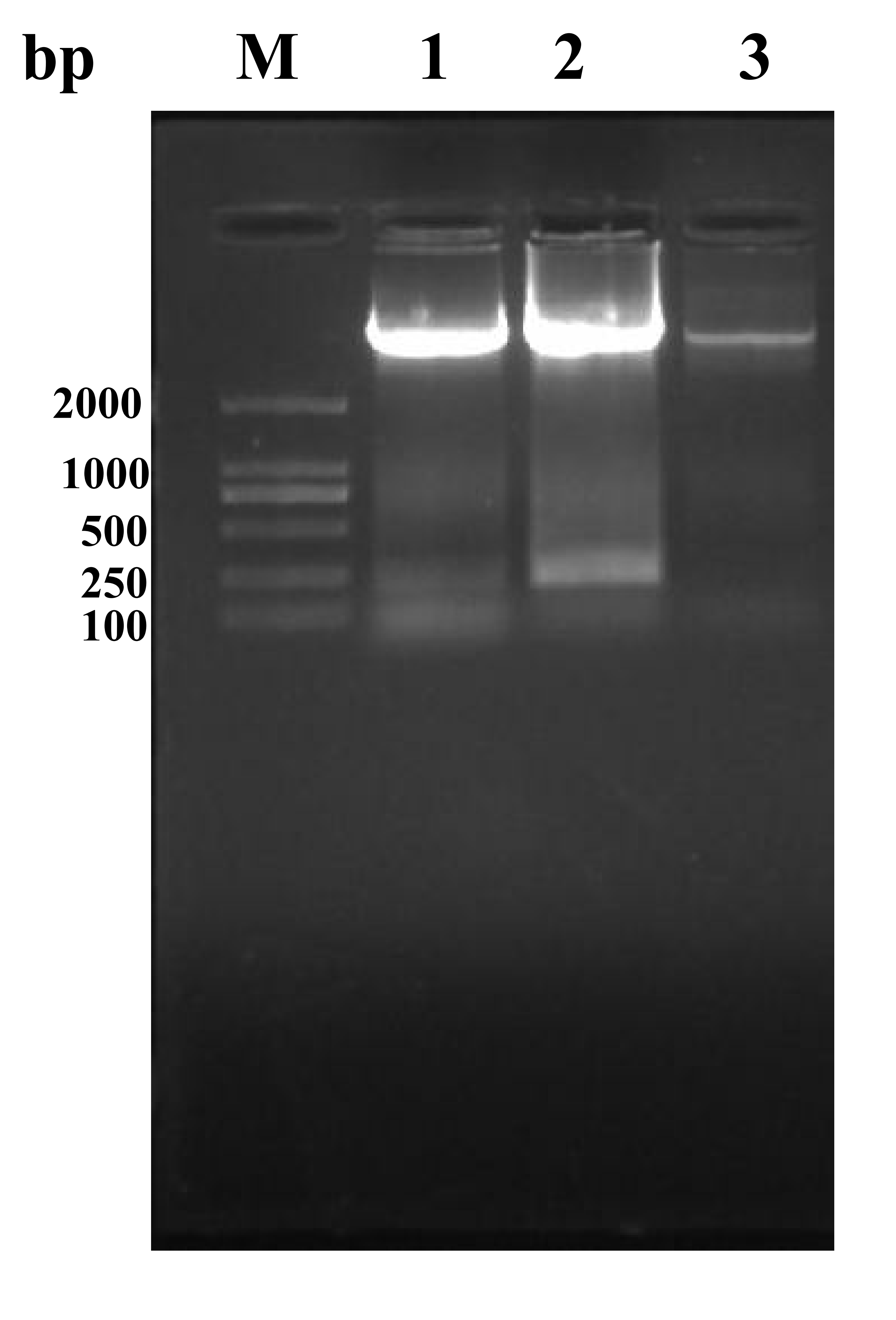


PCR detection. Lane M, DNA marker; lane 1-3, PCR products from linearization product of the vector pETDuet-1.

**Figure S4. The agarose gel electrophoresis analysis for PCR products of *Ec*SstT and *Sc*AAO and universal primer validation of recombinant *E. coli* BL21(DE3)/pETDuet-1-*Sc*aao-*Ec*sstt**


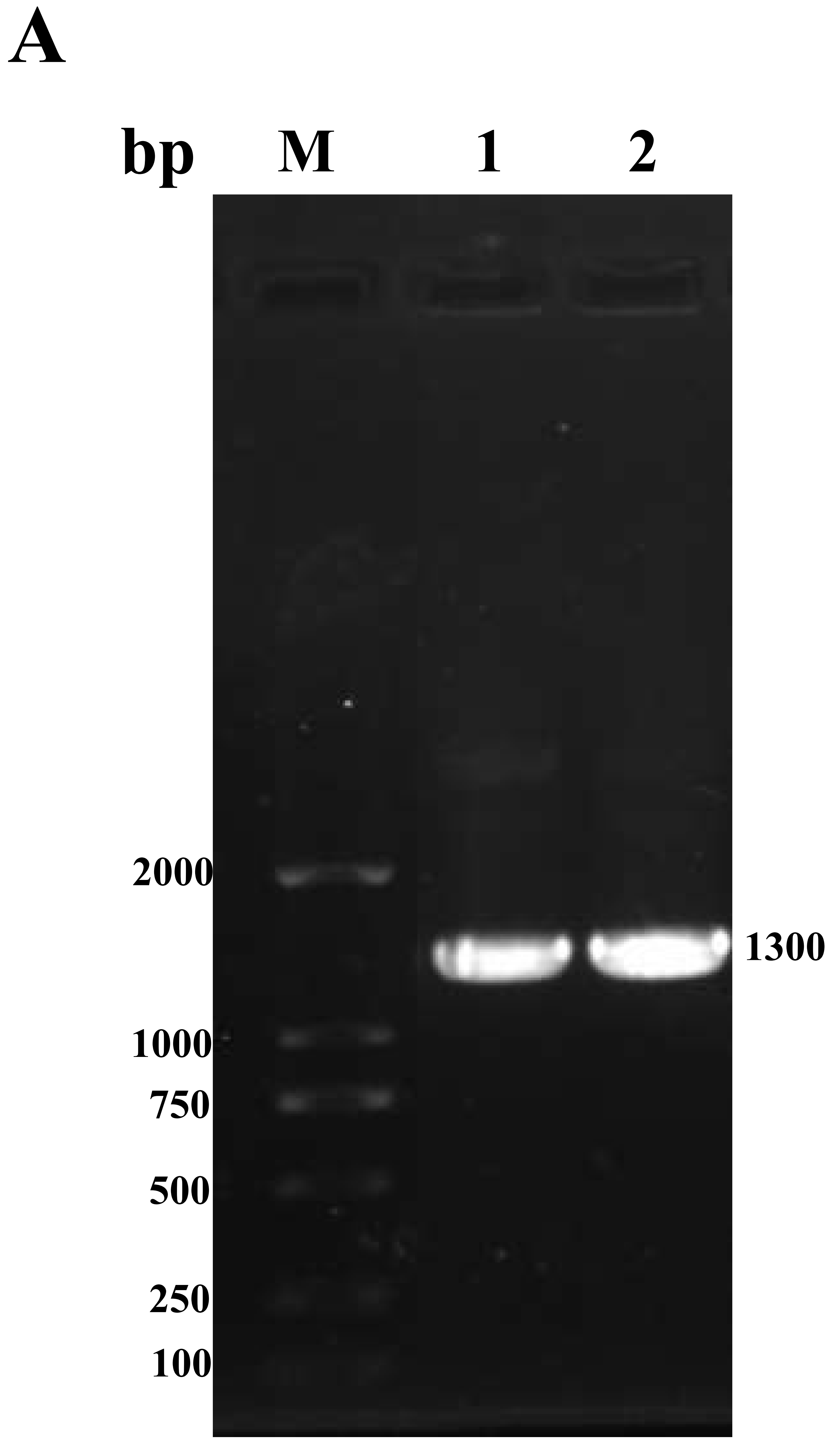

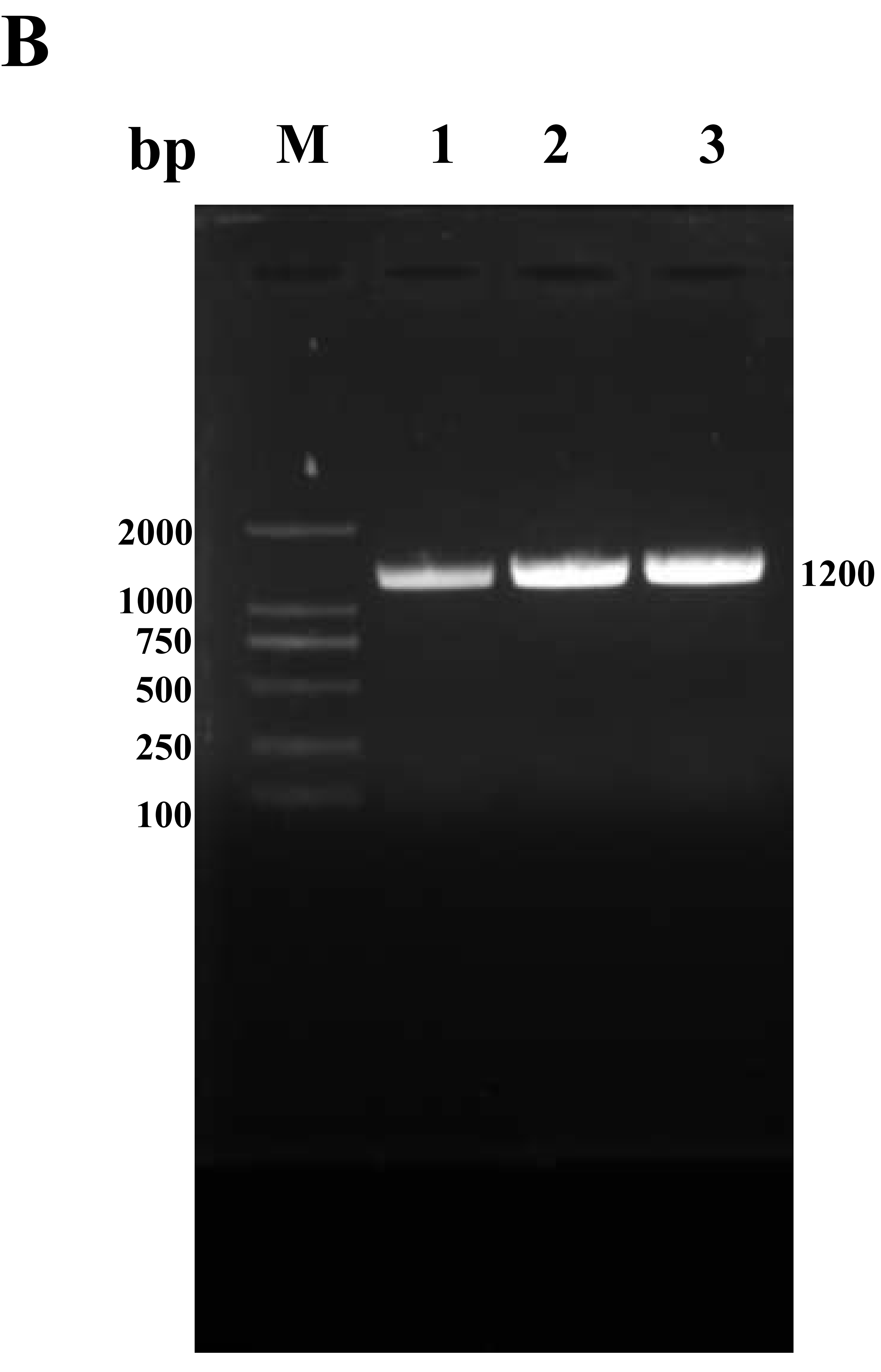

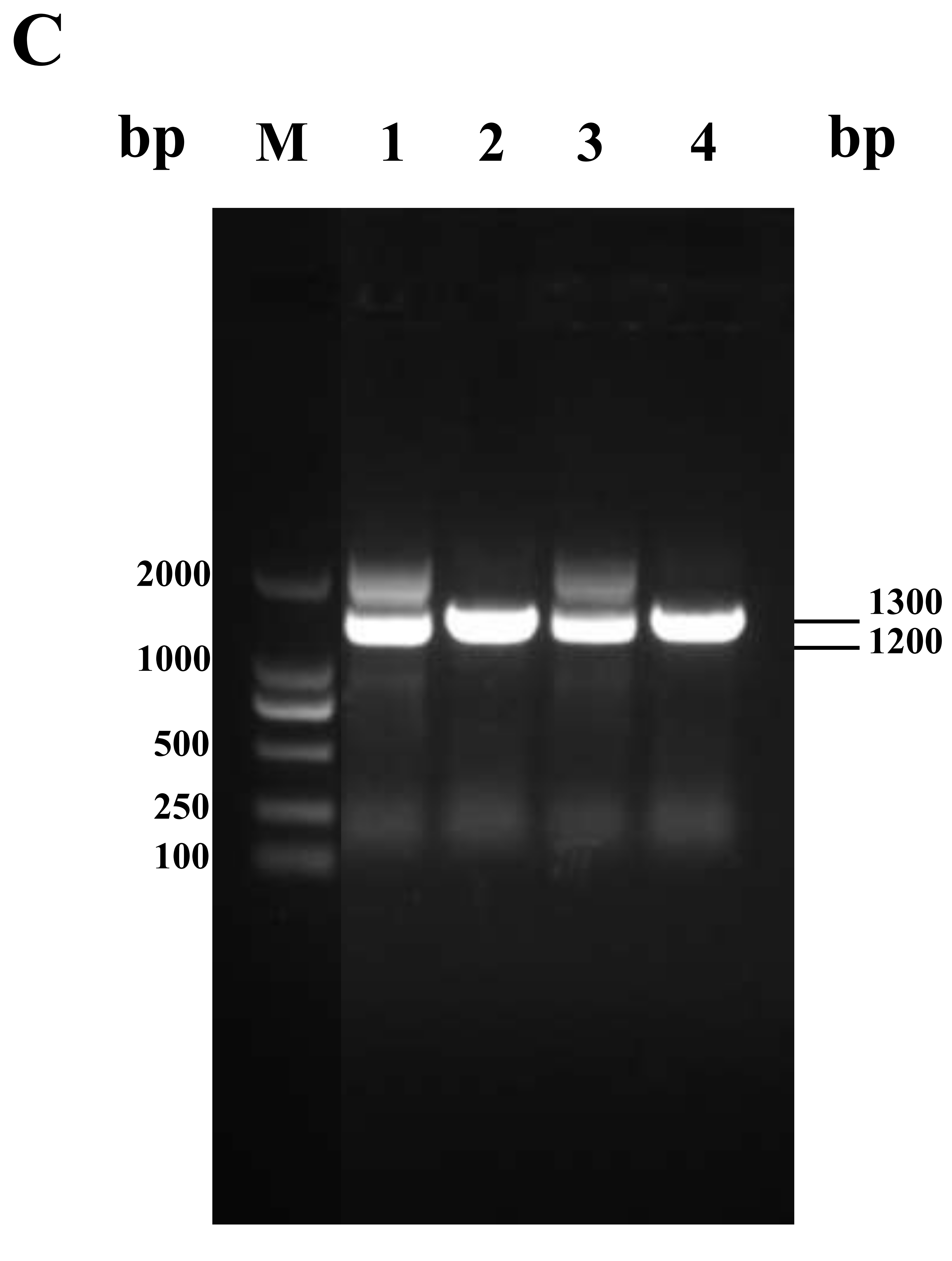


(A) PCR detection. Lane M, DNA marker; lane 1-2, PCR products from different strains of *E. coli* BL21(DE3)/pETDuet-1-*Ec*sstt; (B) PCR detection. Lane M, DNA marker; lane 1-3, PCR products from different strains of *E. coli* BL21(DE3)/pETDuet-1-*Sc*aao;

(C) Universal primer validation *Ec*SstT and *Sc*AAO. Lane M, DNA marker; lane 1 and 3, Universal primer DuetUP_2_ and T_7_ Terminator validation; lane 2 and 4, Universal primer upstream primer and DuetDOWN_1_ validation.

**Figure S5. The universal primer validation of recombinant *E. coli* BL21(DE3)/pACYCDuet-1-*Ec*tdh-*Eh*nox: pETDuet-1-*Sc*aao-*Ec*sstt**


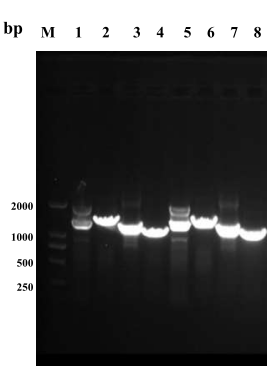


Universal primer validation *Ec*TDH、*Eh*NOX、*Ec*SstT and *Sc*AAO. Lane M, DNA marker; lane 1 and 5, pETDuet-1: Universal primer DuetUP_2_ and T_7_ Terminator validation; lane 2 and 6, pETDuet-1: Universal primer upstream primer and DuetDOWN_1_ validation; lane 3 and 7, pACYCDuet-1: Universal primer DuetUP_2_ and T_7_ Terminator validation; lane 4 and 8, pACYCDuet-1: Universal primer ACYCDuetUP_1_ and DuetDOWN_1_ validation.

**Figure S6. Correlation analysis of 2,5-DMP with peak area.** Prepare 9.1 mM 2,5-DMP stock solution and dilute it to standard solutions of 1.82 mM, 2.27 mM, 3.03 mM, 4.55 mM and 9.10 mM respectively as standard koji of immobilized enzyme. The abscissa is the 2,5-DMP standard concentration, and the ordinate is the peak area.


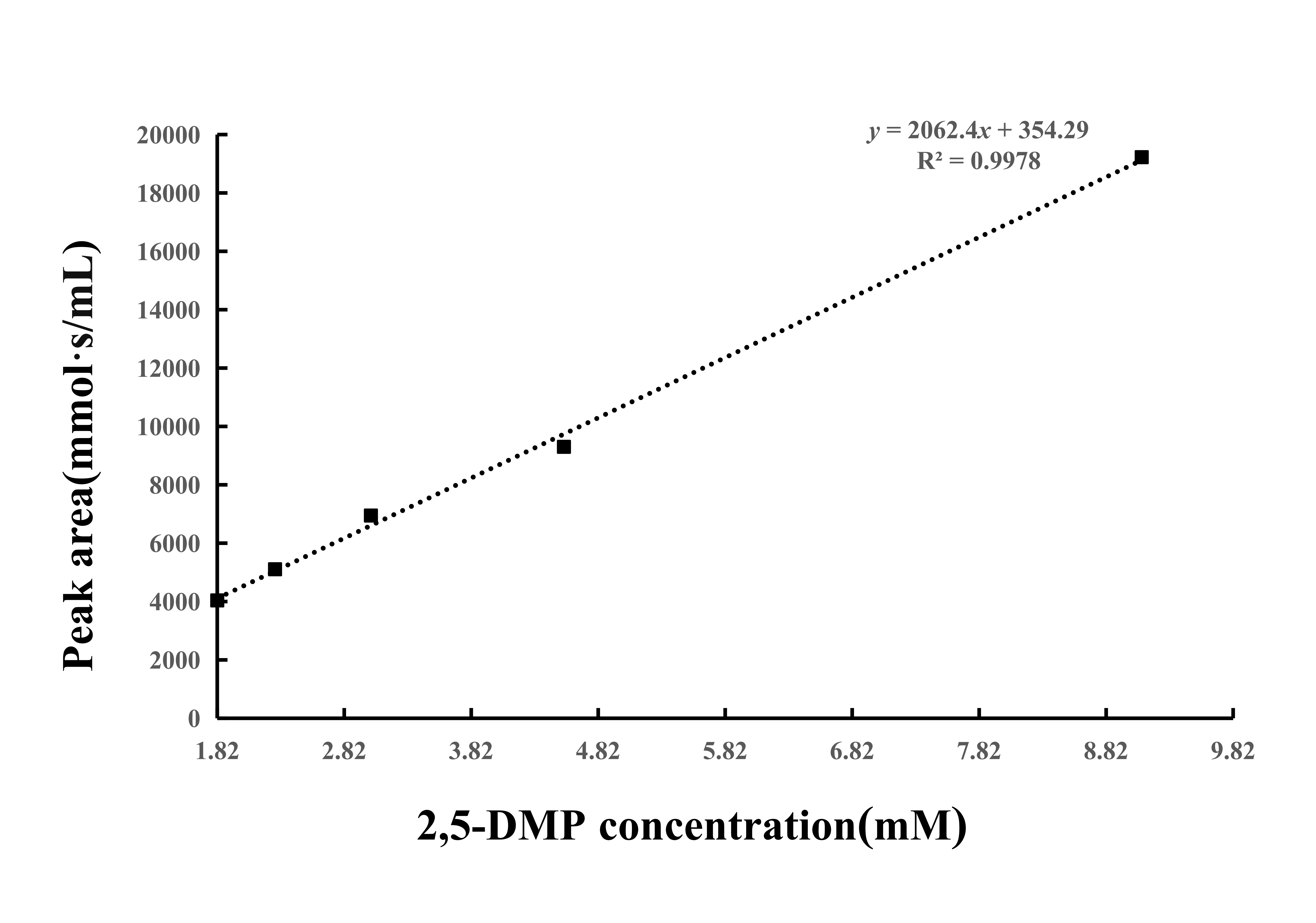


**Figure S7A. HPLC analysis of substrate *L*- threonine standard and product 2,5-DMP.** (A)Retention time of *L*-threonine standard in HPLC.


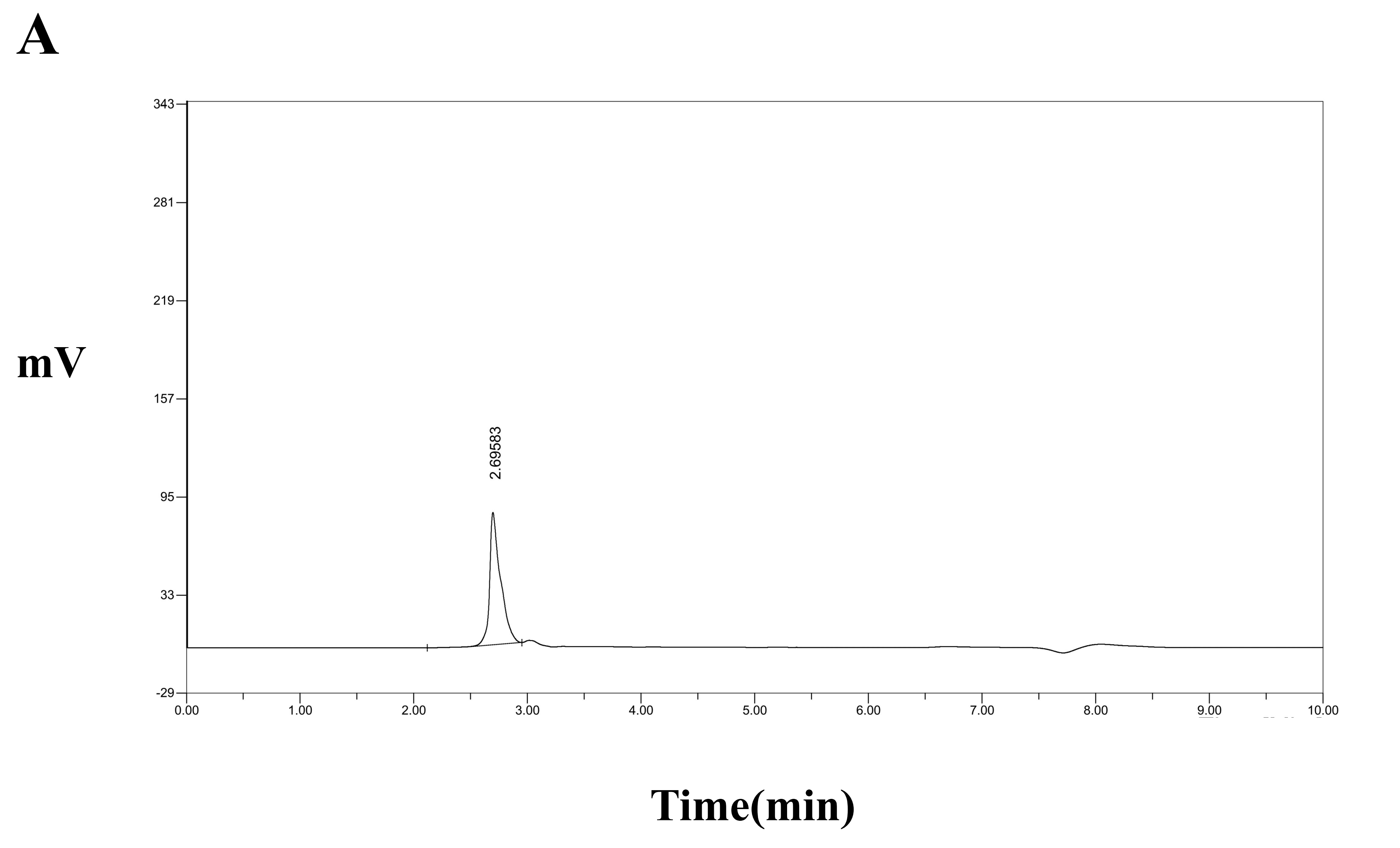


**Figure S7B. HPLC analysis of substrate *L*- threonine standard and product 2,5-DMP.** (B) Retention time of 2,5-DMP standard in HPLC.


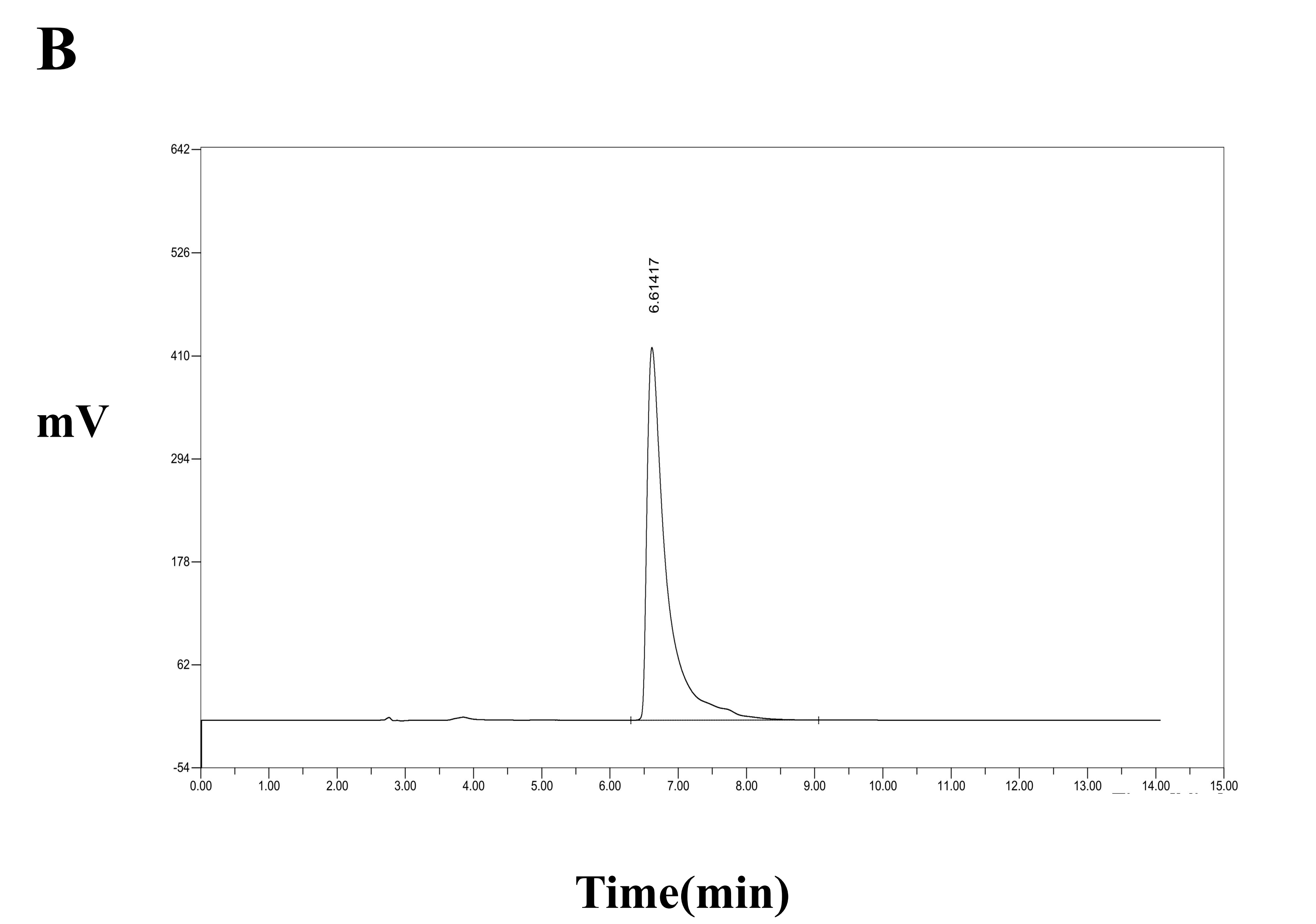


**Figure S8. HPLC analysis catalytic products.**

**mV**

**Time(min)**


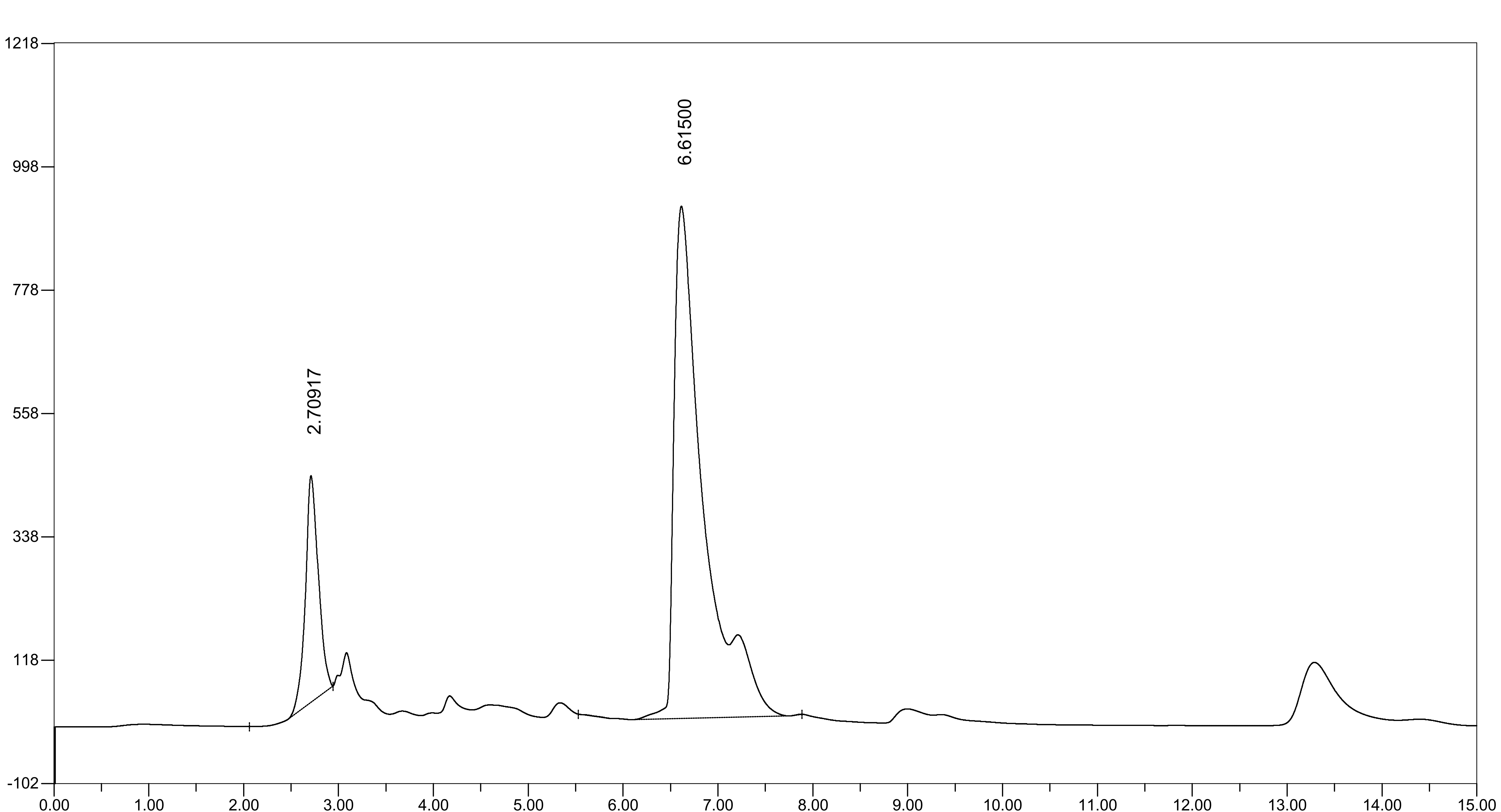


**Figure S9A. NMR and MS plots of products in fermentation broths.**

(A) NMR analysis of products in fermentation broths.

^
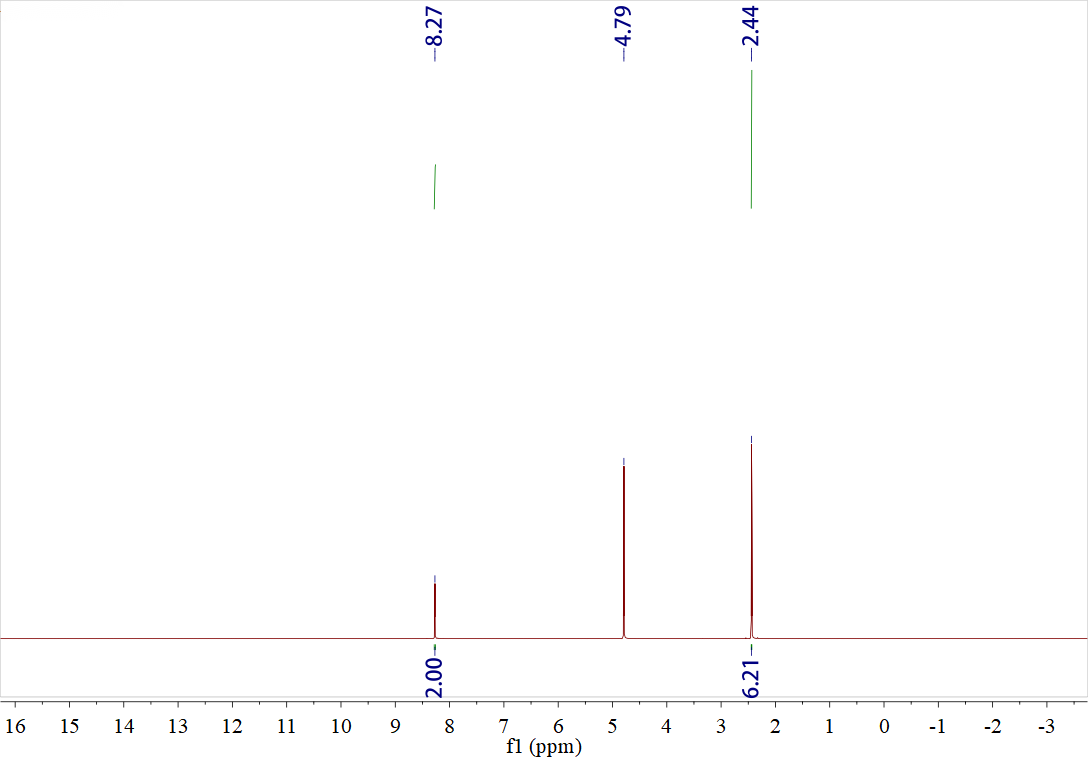
^

^1^H NMR (600 MHz, D_2_O) *δ* : 8.27（s, 2H）,2.44(s, 6H)

**Figure S9B. NMR and MS plots of products in fermentation broths.**

(B) MS analysis of products in fermentation broths.


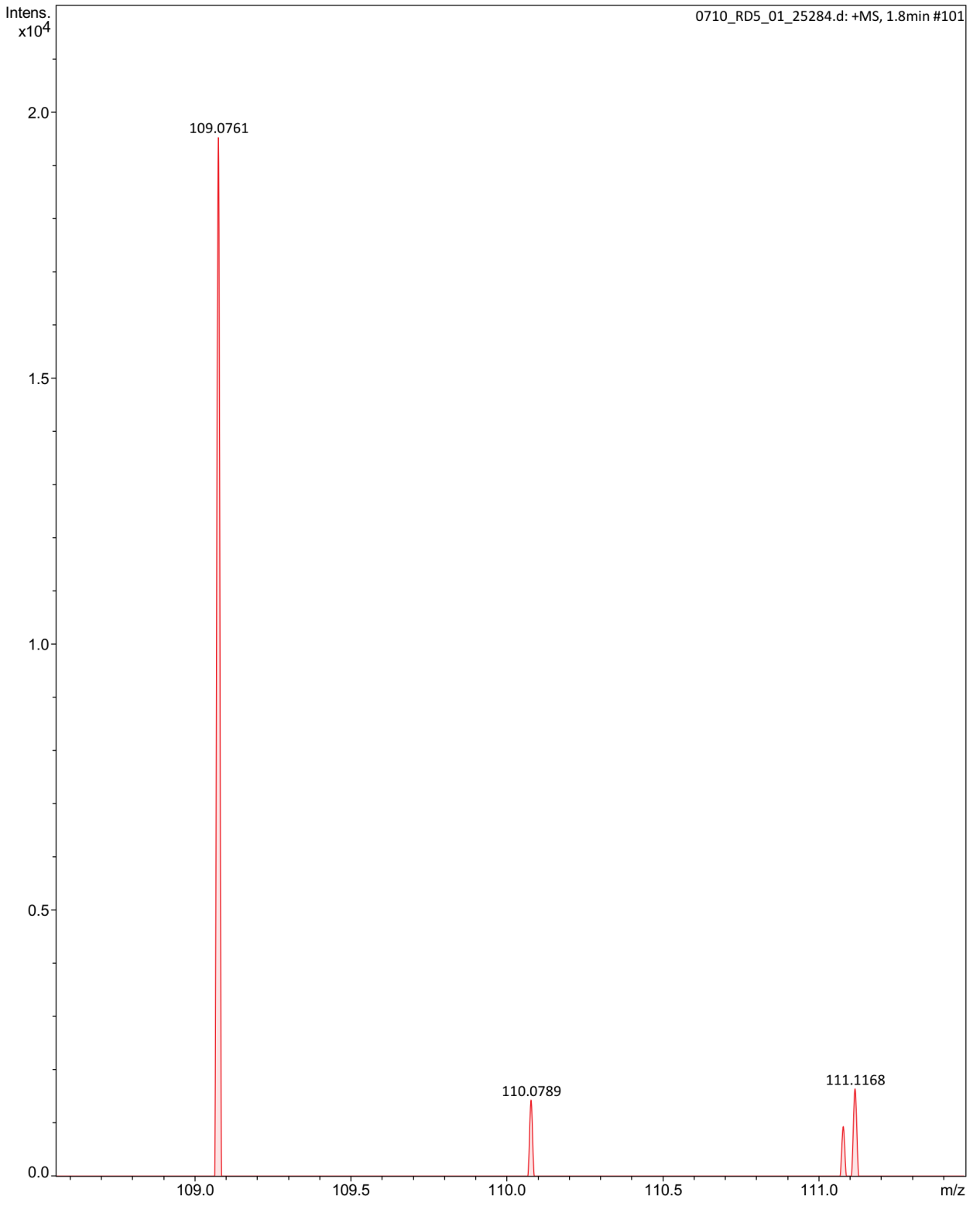


+ESI-HRMS: Calcd for C_6_H_8_N_2_ [M+H]^+^ 109.0760, found: 109.0761.

**Figure S10A. NMR and MS plots of substrates in fermentation broths.**

(A) NMR analysis of substrates in fermentation broths.


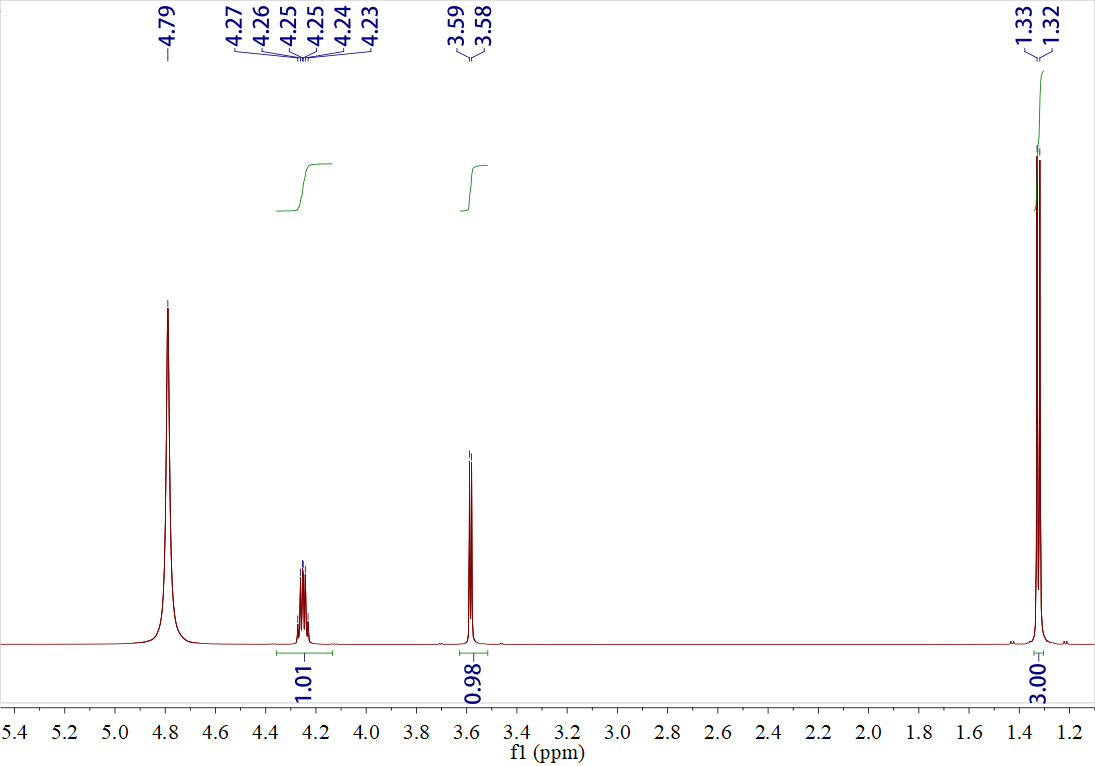


^1^H NMR (600 MHz, D_2_O) *δ* : 4.27-4.23 (td, 1H)，3.59-3.58（d, 1H）,1.33-1.32(d, 3H)

**Figure S10B. NMR and MS plots of substrates in fermentation broths.**

(B) MS analysis of substrates in fermentation broths.


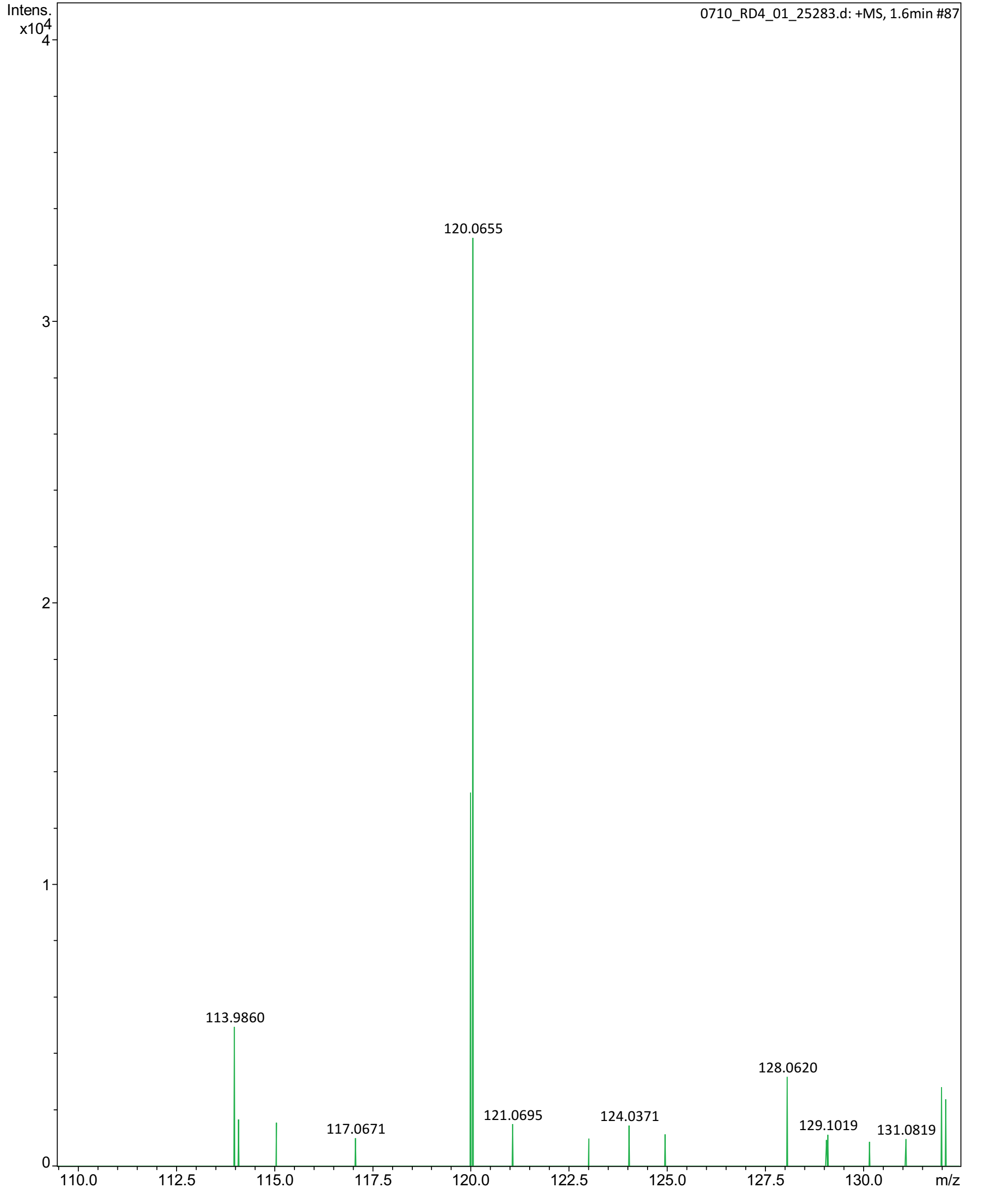


+ESI-HRMS: Calcd for C_4_H_9_NO_3_ [M+H]^+^120.0661, found: 120.0655.

**Figure S11. The results in Figure 4 were analyzed for significance of differences.**

**Figure S12. The results in Figure 5 were analyzed for significance of differences.**

**Figure S13. The results in Figure 6 were analyzed for significance of differences.**

**Figure S14. The results in Figure 7 were analyzed for significance of differences.**

**Table S1. Strains and plasmids used in this study.**

| Strains and plasmids | features | source |
| --- | --- | --- |
| Strains |  |  |
| *E. coli* BL21(DE3) | Clone hosts and expression hosts | This laboratory preserves |
| *Enterococcus hirae* | Clone the host | This laboratory preserves |
| *Streptococcus cristatus* | Clone the host | This laboratory preserves |
| *E. coli* BL21/pACYCDuet-1 | The *E. coli* BL21 strain carries the plasmid pACYCDuet-1 | This study builds |
| *E. coli* BL21/pACYCDuet-1-*Ec*tdh | The *E. coli* BL21 strain carries the plasmid pACYCDuet-1-*Ec*tdh, an exogenous expression of *Ec*TDH | This study builds |
| *E. coli* BL21/pACYCDuet-1-*Ec*tdh-*Eh*nox | The *E. coli* BL21 strain carries exogenous co-expression of the plasmid pACYCDuet-1-*Ec*tdh-*Eh*nox, Co-expression of *Eh*NOX and *Ec*TDH | This study builds |
| *E. coli* BL21/pETDuet-1 | The *E. coli* BL21 strain carries the plasmid pETDuet-1 | This study builds |
| *E. coli* BL21/pETDuet-1-*Sc*aao-*Ec*sstt | The *E. coli* BL21 strain carries exogenous co-expression of the plasmid pETDuet-1-*Sc*aao-*Ec*sstt, Co-expression of *Ec*SstT and *Sc*AAO | This study builds |
| *E. coli* BL21/pACYCDuet-1-*Ec*tdh-*Eh*nox: pETDuet-1-*Sc*aao-*Ec*sstt | The *E. coli* BL21 strain carries exogenous co-expression of the double plasmid pACYCDuet-1-*Ec*tdh-*Eh*nox and pETDuet-1-*Sc*aao-*Ec*sstt, Co-expression of *Ec*TDH、*Eh*NOX、*Ec*SstT and *Sc*AAO | This study builds |
| plasmid |  |  |
| pACYCDuet-1 | *E. coli* expression vectors | This study builds |
| pACYCDuet-1-*Ec*tdh | Exogenously expressed recombinant plasmid of TDH derived from *E. coli* BL21, plasmid pACYCDuet-1 carries the *Ec*TDH gene (Cm^r^) derived from *E. coli* BL21 | This study builds |
| pACYCDuet-1-*Ec*tdh-*Eh*nox | Exogenous co-expression recombinant plasmids derived from TDH from *E. coli* BL21 and NOX from *Enterococcus hirae*, plasmid pACYCDuet-1 carries the *Ec*TDH gene derived from *E. coli* BL21 and the *Eh*NOX gene (Cm^r^) derived from *Enterococcus hirae* | This study builds |
| pETDuet-1 | *E. coli* expression vectors | This study builds |
| pETDuet-1-*Sc*aao-*Ec*sstt | Exogenous co-expression recombinant plasmids derived from SstT from *E. coli* BL21 and AAO from *Streptococcus cristatus*, plasmid pETDuet-1 carries the *Ec*SstT gene derived from *E. coli* BL21 and the *Sc*AAO gene (Amp^r^) derived from *Streptococcus cristatus* | This study builds |
| pACYCDuet-1-*Ec*tdh-*Eh*nox: pETDuet-1-*Sc*aao-*Ec*sstt | Exogenous co-expression recombinant plasmids derived from TDH and SstT from *E. coli* BL21 and NOX from *Enterococcus hirae* and AAO from *Streptococcus cristatus*, plasmid pACYCDuet-1 carries the *Ec*TDH gene derived from *E. coli* BL21 and the *Eh*NOX gene (Cm^r^) derived from *Enterococcus hirae* and plasmid pETDuet-1 carries the *Ec*SstT gene derived from *E. coli* BL21 and the *Sc*AAO gene (Amp^r^) derived from *Streptococcus cristatus* | This study builds |

**Table S2. Primers used in this study.**

| Primer name | Primer sequences（5’→3’） | description |
| --- | --- | --- |
| *Ec*tdh-F | CGCGGATCCGATGAAAGCGTTATCCAAACTG | Amplification of the gene fragment *Ec*TDH for the construction of the plasmid pACYCDuet-1-*Ectdh* |
| *Ec*tdh-R | CCCAAGCTTTTAATCCCAGCTCAGAATAAC |  |
| *Eh*nox-F | GGAATTCCATATGAAAGTAGTAGTAATTGGT | Amplification of the gene fragment *Eh*NOX for the construction of the plasmid pACYCDuet-1-*Ectdh*-*Ehnox* |
| *Eh*nox-R | CCGCTCGAGTTATTGTTTTGTTTCCATTTC |  |
| pETDuet-1-F_1_ | GTGGTGATGATGGTGATGGCTG | The vector pETDuet-1 is linearized and contains a homologous arm of the *Ec*SstT gene |
| pETDuet-1-R_1_ | AGCCAGGATCCGAATTCGAGCT |  |
| pETDuet-1-F_2_ | CATATGTATATCTCCTTCTTATACTTAAC | The vector pETDuet-1 is linearized and contains a homologous arm of the *Sc*AAO gene |
| pETDuet-1-R_2_ | AGCCTGCACTATGATTAGGCAGATCTCAATTGGATATCG |  |
| *Ec*sstt-F | CATCACCATCATCACCACATGACTACGCAACGTTCACCG | Amplification of the gene fragment *Ec*SstT for the construction of the plasmid pETDuet-1-*Ecsstt* |
| *Ec*sstt-R | CGAATTCGGATCCTGGCTTTAATTACGCAGGGCGCTATT |  |
| *Sc*aao-F | GAAGGAGATATACATATGATGAATCACTTTGATACAATT | Amplification of the gene fragment *Sc*AAO for the construction of the plasmid pETDuet-1*-Sc*aao-*Ec*sstt |
| *Sc*aao-R | CTAATCATAGTGCAGGCTGCC |  |

**Table S3. HPLC gradient elution of the product 2,5-DMP.**

| Number | Time（min） | Mobile phase A（%） | Mobile phase B（%） |
| --- | --- | --- | --- |
| 1 | 0.00 | 69 | 31 |
| 2 | 3.00 | 31 | 69 |
| 3 | 3.01 | 69 | 31 |
| 4 | 10.00 | 69 | 31 |
